# Mechanisms of Resistance to PARPi in Pancreatic Ductal Adenocarcinoma

**DOI:** 10.1111/jcmm.70816

**Published:** 2025-08-28

**Authors:** Jojanneke Stoof, Charlotte Andrieu, Fiona O'Connell, Jacintha O'Sullivan, Maeve A. Lowery, Naomi Walsh

**Affiliations:** ^1^ Trinity St. James Cancer Institute Trinity College Dublin Dublin Ireland; ^2^ School of Biotechnology, Life Sciences Institute Dublin City University Dublin Ireland

**Keywords:** cisplatin, metabolism, pancreatic ductal adenocarcinoma, PARP inhibitor, resistance

## Abstract

Pancreatic ductal adenocarcinoma (PDAC) is a highly fatal disease with limited treatment options. PARP inhibitors (PARPi) have shown promise in treating PDAC with homologous recombination deficiency (HRD), but rapid acquisition of resistance limits their efficacy. Our objective is to investigate mechanisms of resistance to PARPi in *BRCA2*‐mutant PDAC cells and identify potential therapeutic targets to modulate this resistance. We developed olaparib‐ and talazoparib‐resistant Capan‐1 cell lines and characterised their resistance profiles using viability assays, RNA sequencing and metabolomic profiling. We also developed a cisplatin‐resistant Capan‐1 cell line to compare resistance mechanisms between PARPi and platinum agents. Both olaparib‐ and talazoparib‐resistant cells showed cross‐resistance to other PARPi and oxaliplatin, but not to gemcitabine or 5‐FU. Talazoparib‐resistant cells exhibited a similar resistance profile to cisplatin‐resistant cells, including decreased PARP1 expression and altered metabolomic profiles. RNA sequencing and metabolomic profiling revealed significant enrichment of metabolic pathways, including oxidative phosphorylation and glycolysis, in resistant cells. Our study highlights the complexity of resistance mechanisms to PARPi in PDAC and identifies potential therapeutic targets in metabolism. The differences in the resistance profiles between olaparib and talazoparib suggest that PARP‐trapping potency may play a role in resistance development. Further research is needed to validate these findings and explore novel therapeutic strategies to overcome resistance to PARPi in PDAC.

**Abbreviations**
5‐FU: 5‐fluorouracil
ATCC: American Tissue Culture Collection
CNA: copy number alteration
DSB: double‐strand break
ECAR: extracellular acidification rate
FBS: fetal bovine serum
HRD: homologous recombination deficiency
LOH: loss of heterozygosity
NHEJ: non‐homologous end‐joining
OCR: oxygen consumption rate
OxPhos: oxidative phosphorylation
PAR: polyADP ribose
PARPi: poly (ADP‐ribose) polymerase inhibitor
PBS: phosphate buffered saline
PDAC: Pancreatic ductal adenocarcinoma
SRC: spare respiratory capacity
WES: whole exome sequencing

## 1 Introduction

Pancreatic ductal adenocarcinoma (PDAC) is the predominant form of pancreatic cancer and is a highly fatal disease with a dismal prognosis. Curative treatment is limited to patients with resectable disease; however, recurrent disease is common and the majority of patients are diagnosed at an advanced stage. Current standard of care treatment for advanced PDAC consists of systemic chemotherapy, such as FOLFIRINOX, NALIRIFOX, or a combination of gemcitabine with nab‐paclitaxel [1, 2].

PARP1 is the predominant member of the poly (ADP‐ribose) polymerase (PARP) family and uses NAD^+^ to add single or polyADP ribose (PAR) moieties onto itself and target proteins to regulate protein function [3, 4, 5]. While PARP1 has been described to play a role in both single‐strand and double‐strand break repair, metabolism, regulation of apoptosis and cell death, it is primarily involved in promoting single‐strand break (SSB) repair through the base excision repair pathway. Using its DNA‐binding domain, PARP1 recognises SSBs, which activates auto‐PARylation, which in turn stimulates PARylation of target proteins and recruitment of scaffolding protein XRCC1.

Less than 5% of PDAC have mutations in BRCA1 (1.1%), BRCA2 (3.1%), or PALB2 (0.6%) [6]. These genes are essential for homologous recombination, and loss of function results in homologous recombination deficiency (HRD). HRD has been shown to sensitise to platinum agents but is also synthetic lethal with PARP inhibitors (PARPi) [4, 7, 8]. Several PARPi, including olaparib, talazoparib, niraparib and rucaparib, have received FDA approval for solid tumours with HRD, although currently only olaparib is approved for PDAC. These PARPi are nicotinamide analogs that compete with the NAD^+^ binding domain of PARP1, which blocks its PARylation function and traps the protein at SSBs. This not only leads to the accumulation of SSBs but can also impair replication for progression during the S phase of the cell cycle, which in turn can lead to replication fork stalling or collapse and the formation of double‐strand breaks (DSB). In HRD cells, DSB repair is impaired and therefore DSBs will be either repaired by NHEJ, which has a low fidelity and leaves insertions/deletions at the location of the break, or the breaks will accumulate, which eventually results in cell death [7, 8].

Like with many cancers therapeutic agents, cancer cells rapidly acquire resistance to PARPi. A key resistance mechanism to PARPi in HRD tumours that has been observed in in vitro studies and in patients is reversion mutation of BRCA1/2, leading to HR restoration [9, 10]. Reversion mutations typically do not fully restore the entire gene sequence, but do partially restore protein function. However, reversion mutations are not found in all HRD tumours with acquired resistance, indicating that additional resistance mechanisms are involved [11]. Two additional processes identified to play a role in PARPi resistance in vitro are increased drug elimination (caused by elevated levels of drug export pumps) and loss of PARP1 expression [12, 13], although these mechanisms also do not account for the in vitro resistance [14].

Therefore, the objective of the present study was to create isogenic in vitro models of olaparib and talazoparib‐resistant PDAC derived from the *BRCA2*‐mutant cell line Capan‐1. We subsequently characterised the parental and resistant models with regard to (a) resistance and cross‐resistance to commonly used chemotherapies, (b) previously described resistance mechanisms and (c) RNA, DNA and metabolomic profiling.

## 2 Material and Methods

### 2.1 Cell Culture

The human pancreatic cell line Capan‐1 was obtained from the American Type Culture Collection (ATCC) and was maintained in DMEM medium (#41965‐039, Gibco) supplemented with 10% FBS (#F7524, Sigma‐Aldrich) and 1% Glutamax (#35050038, BioSciences). Cells were propagated in a humidified atmosphere with 5% CO_2_ at 37°C. Mycoplasma testing was regularly performed using the MycoStrip detection kit (#rep‐mysnc‐50, InvivoGen).

### 2.2 Generation of PARPi Resistant Cell Lines

To generate PARP inhibitor resistant cells, Capan‐1 cells were treated with 450 nM olaparib (#17281212, FisherScientific) or talazoparib (#S7048, Selleckchem) for 14 days, given a drug break of 45 days, followed by continuous exposure at an increasing concentration from 50 to 75 or 100 nM for olaparib and talazoparib respectively. Cisplatin resistant cells were generated similarly, with an initial concentration of 75 nM, and continous exposure at increasing concentration from 20 to 50 nM. After confirmation of resistance, cells were maintained in 75 nM drug PARPi or 50 nM cisplatin.

### 2.3 Proliferation Assays

5 × 10^3^ cells/well were plated in 96‐well plates. After 24 h, media containing olaparib, talazoparib, cisplatin (#CAYM13119‐50, VWR), gemcitabine (#S1714, Selleckchem), oxaliplatin (#S1224, Selleckchem), or SN38 (#A12011, CliniSciences) was added and cells were allowed to proliferate for 7 days. For combination treatment with FOLFIRINOX, drugs were combined in a ratio similar to what is given to patients, where 100% FOLFIRINOX was set at 0.428 μM folinic acid, 34.4 μM 5‐fluorouracil, 0.4 μM SN‐38 and 0.32 μM oxaliplatin [15]. Cell survival was determined by PrestoBlue (#P50201, ThermoFisher) staining. 20 μL PrestoBlue was added to each well, cells were incubated for 3 h, and fluorescence was measured on a plate reader (Infinite 200, Tecan) using Gen4 software.

### 2.4 DNA and RNA Extraction and Sequencing

DNA and RNA were extracted from Capan‐1 parental and resistant cells using the DNA/RNA AllPrep kit (#80204, Qiagen), *n* = 1. DNA and RNA concentrations were quantified using Nanodrop and samples were sequenced by Novogene using the Illumina NovaSeq platform. Library preparation (polyA enrichment for RNA and SureSelect Human All Exon V6 for DNA) and sequencing were performed by Novogene. Samples were sequenced using the Illumina NovaSeq platform with 100× coverage.

### 2.5 Whole Exome Sequencing Analysis

DNA sequencing data was analysed using the SomaSnake pipeline (v1.3) [16]. In short, reads were trimmed using Sickle and mapped by Burrows‐Wheeler Aligner on human genome build 38. Genome Analysis Toolkit (GATK) was used to remove duplicates, recalibrate mapped reads and apply base quality score recalibration [17]. Evaluation and visualisation of coverage depth was performed by Mosdepth, Sambamba and Samtools [18, 19, 20]. Variant calling, estimation of contamination and detection of false positive SNPs were performed using the GATK4 tools Mutect2, CalculateContamination and FilterMutectCalls, respectively [21]. Bcftools and vcfintersect were used to filter and format output. Control‐FREEC was used to detect copy number alterations (CNA), B‐allele frequency (BAF) and loss of heterozygosity (LOH) [22]. Somatic variants were annotated using the Variant Effect Predictor tool and intersectBed [23, 24].

### 2.6 Transcriptomic Analysis

Transcriptomic analysis was performed using the public server at usegalaxy.org [25]. Quality control was performed using FastQC and multiQC. Adapter sequences present in a fraction of the reads were removed using Cutadapt (sequence AGATCGGAAGAG, filter minimum length: 20, quality cutoff: 20) and trimmed reads were mapped onto the human reference genome (Hg38) using HISAT2 [26]. Count reads were mapped to genes with FeatureCounts. To determine enriched pathways, differential expression analysis was performed in Omicsbox [27] using the NOISeq package with the parameters in table (CPM filter: 0.5; normalisation: TMM; number of simulated replicates: 5; size of simulated replicates: 0.2; variability: 0.02). The output was then used to perform Gene Set Enrichment Analysis at usegalaxy.org [28].

### 2.7 Metabolic Analysis

ATP flux and mitochondrial stress were evaluated using the Seahorse Xfe24 analyser. Cells were seeded in quadruplicate in a 24‐well Seahorse plate at 1.8 × 10^3^ cells/well. After 48 h, the media was removed and the cells were washed with Seahorse media (media, 10 mM glucose, 1 mM sodium pyruvate, 2 mM l‐glutamine). After washing, 500 μL Seahorse media was left in the wells and the cells were incubated at 37°C in a non‐CO2 incubator for 1 h. Oxygen consumption rates (OCR) and extracellular acidification rates (ECAR) were measured before and after treatment with 0.9 μM oligomycin, 2 μM FCCP and 2 μM antimycin according to the Seahorse ATP flux and MitoStress assay protocols (#103015‐100, Agilent Technologies). Measurements were normalised to cell number using a crystal violet assay. Cells were fixed in 1% glutaraldehyde in PBS (#340855, Sigma‐Aldrich) for 15 min at room temperature. The fixative was removed, and the cells were washed with PBS. The cells were stained with 50 μL crystal violet (1% in water) for 30 min at room temperature. The dye was removed, and the cells were washed with UHP water and allowed to air dry overnight. The dye was solubilised with Triton X (1% in PBS) on a shaker at 400 rpm for 1 h. Absorbance was read at 595 nm using the Promega Glomax explorer.

### 2.8 Statistical Analysis

Statistical analyses were performed using GraphPad Prism version 10.4.1 for Windows (GraphPad Software, Boston, USA). Viability curves were plotted with a non‐linear fit (three parameters). IC_50_ values were extrapolated from individual replicates and compared to parental IC_50_ values using an unpaired Student's *t*‐test. Statistical analysis of the Seahorse assay was performed by one‐way ANOVA followed by Dunnett's multiple comparisons test with the parental cells set as the control. Data are shown as means ± standard deviation, and a *p*‐value < 0.05 was considered statistically significant.

## 3 Results

### 3.1 In Vitro Modelling of PARP Inhibitor Resistance

**FIGURE 1:**
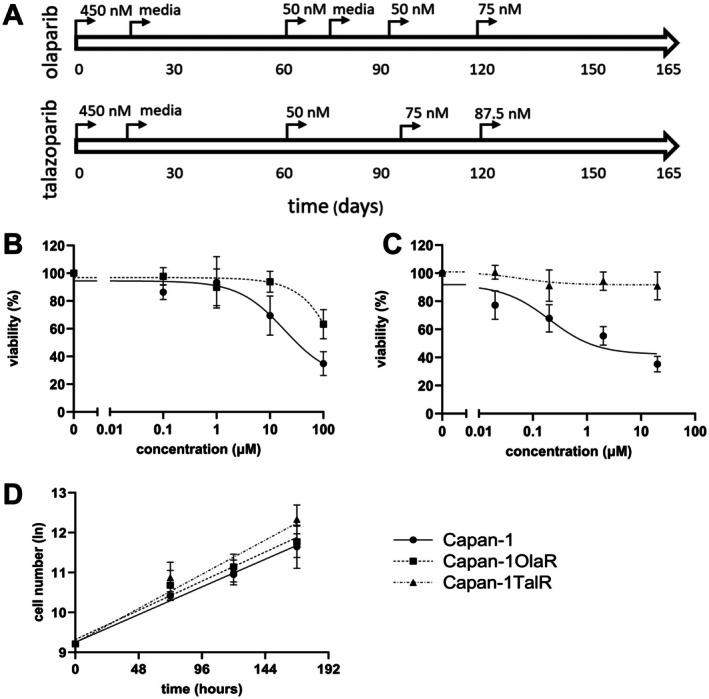
Establishment of olaparib and talazoparib‐resistant Capan‐1 cells. (A) treatment schedule. (B, C) viability assay in parental and resistant cells after treatment with olaparib (B) or talazoparib (C) for 7 days as measured by PrestoBlue staining. (D) Determination of doubling time of parental and resistant cells. Cells were seeded in a 12‐well plate, trypsinised, and counted after 3, 5 and 7 days. The cell number was ln transformed to plot a linear growth curve and doubling time was calculated using nonlinear fit. Error bars indicate standard deviation (*n* = 3).

To analyse the mechanism of PARP inhibitor resistance in pancreatic cancer cells with *BRCA2* deficiency, we developed olaparib and talazoparib resistant Capan‐1 cells (Figure 1A). Cells were initially exposed to 450 nM PARPi which is approximately the IC_10_ and IC_30_ concentration for olaparib and talazoparib, respectively (Figure 1B,C). We noticed induction of senescence after two weeks and gave the cells a drug break to recover, after which treatment was restarted at a lower concentration. After 165‐day exposure, induction of resistance was confirmed by a 7‐day viability assay. Both PARPi treated cell lines showed at least a 3‐fold increase in IC_50_ concentration and were growing well in drugged media and were therefore considered resistant. The olaparib‐treated cells (Capan‐1OlaR) gained over a 3.1‐fold increase in relative resistance toward olaparib compared to the parental Capan‐1 cells (Capan‐1OlaR IC_50_: > 100 μM, Capan‐1 IC_50_: 32 μM) (Figure 1B). The talazoparib‐treated cells (Capan‐1TalR) gained more than an 18‐fold increase in relative resistance toward talazoparib compared to the parental Capan‐1 cells (Capan‐1TalR IC_50_: > 20 μM, Capan‐1 IC_50_: 1.1 μM) (Figure 1C). To determine whether the development of resistance affected the proliferation rate of the Capan‐1 cells, the doubling time was measured for each of the cell lines (Figure 1D). Respective doubling times showed no significant difference between the parental and Capan‐1Ola (*p* = 0.56) but reduced doubling time for Capan‐1TalR was observed (*p* = 0.02): Capan‐1 2.0 days (95% CI 1.74–2.36), Capan‐1OlaR 1.90 days (95% CI 1.61–2.32) and Capan‐1TalR 1.62 days (95% CI 1.37–1.91).

### 3.2 Cross‐Resistance to Clinically Relevant Therapies

**FIGURE 2:**
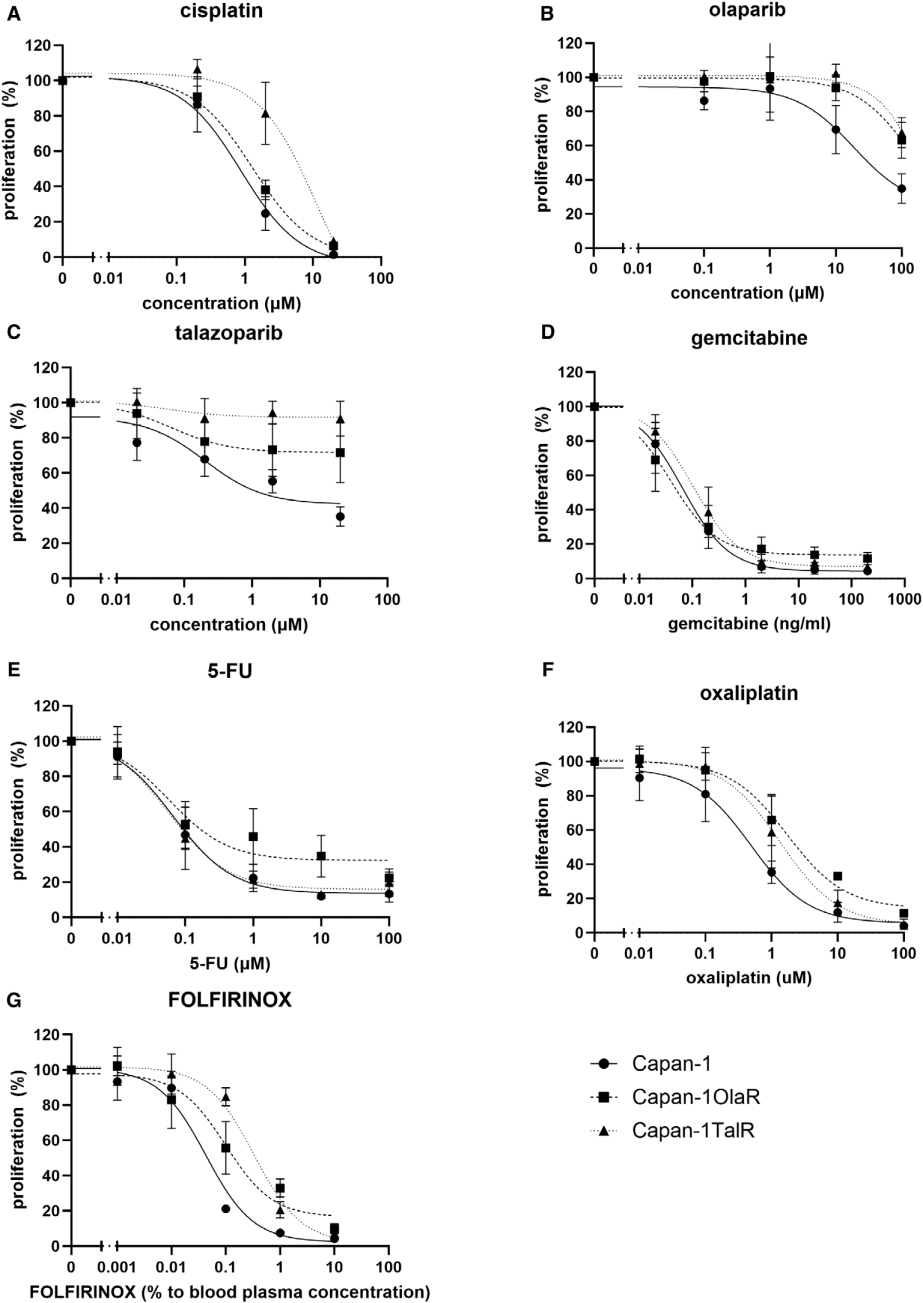
Sensitivity of parental and PARPi resistant Capan‐1 cells to common systemic treatments for PDAC. Viability was measured using PrestoBlue and normalised against untreated control after 7 days of treatment. (A) cisplatin, (B) olaparib, (C) talazoparib, (D) gemcitabine, (E) 5‐FU, (F) oxaliplatin, (G) FOLFIRINOX. FOLFIRINOX is a combination treatment consisting of folinic acid, 5‐fluorouracil, irinotecan and oxaliplatin. Drugs were combined in a ratio similar to what is given to patients, where 100% FOLFIRINOX was set at 0.428 μM folinic acid, 34.4 μM 5‐fluorouracil, 0.4 μM SN‐38 (active compound of irinotecan) and 0.32 μM oxaliplatin [15]. The maximum concentration (100%) is equal to 0.428 μM folinic acid, 34.4 μM 5‐fluorouracil, 0.4 μM SN‐38 (active compound of irinotecan) and 0.32 μM oxaliplatin.

We evaluated if olaparib and talazoparib resistant Capan‐1 exhibited cross‐resistance to other clinically relevant therapies and observed varying levels of sensitivity (Figure 2, Table S1). Both Capan‐1OlaR and Capan‐1TalR exhibited significant cross‐resistance to the other PARPi, as well as to oxaliplatin. Additionally, Capan‐1TalR exhibited significant cross‐resistance to cisplatin and FOLFIRINOX. No significant cross‐resistance was observed for either cell line to gemcitabine or 5‐FU.

### 3.3 Investigation of Known Resistance Mechanisms

Previously, reversal mutation of BRCA2, loss of PARP1 protein expression, and overexpression of drug efflux pumps ABCB1 (p‐glycoprotein) and ABCG2 (BCRP) have been linked to acquired resistance to PAPRi in BRCA2 mutant cancer [9, 12, 13, 29, 30]. Whole exome sequencing of the parental and resistant Capan‐1 cells found that all three cell lines had maintained the c.5946del that results in the truncated protein (p.S1982Rfs*22). Additionally, we detected no novel mutations in other homologous recombination repair genes in the resistant cells.

**FIGURE 3:**
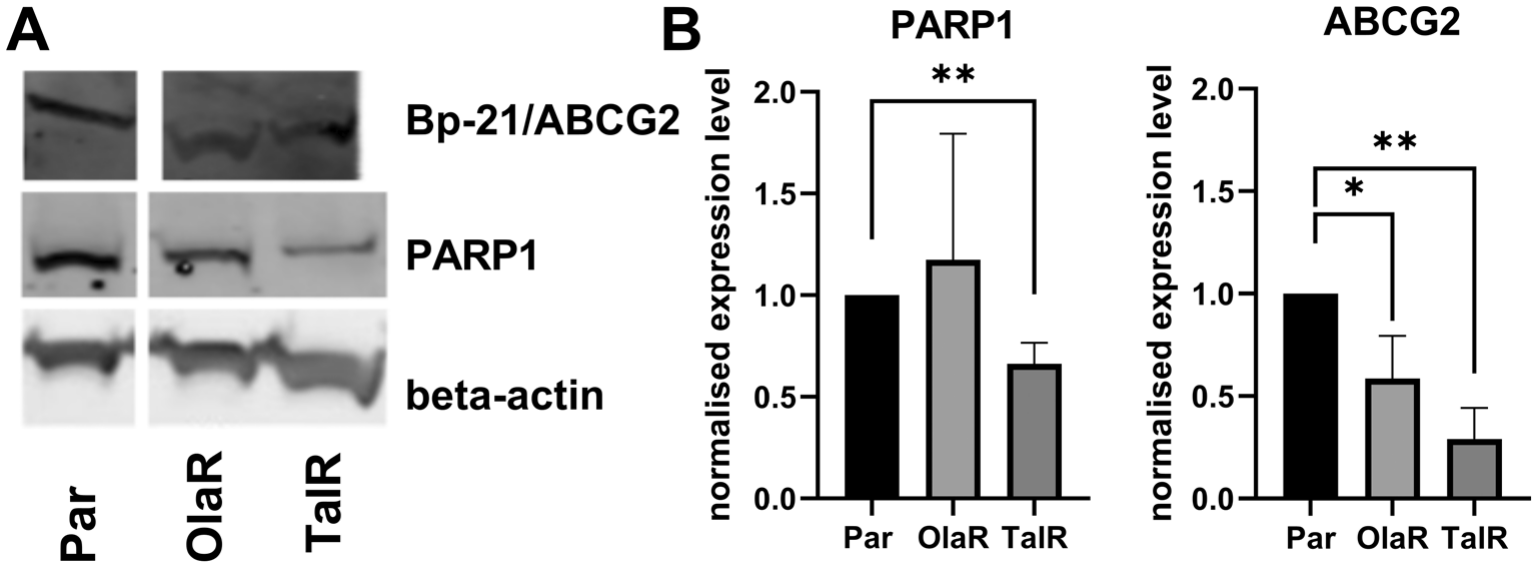
Expression of PARP1 and ABCG2 in Capan‐1 by Western blot. (A) visualisation of ABCG2 and PARP1 protein expression. (B) densitometry analysis of ABCG2 and PARP1 protein expression. Expression was quantified using ImageJ and corrected against the loading control beta‐actin and then normalised against the parental cells. Student's *t*‐test was used to identify statistical differences between each resistant cell line and the parental cell line. Abbreviations: Capan‐1 parental (PAR), Capan‐1OlaR (OlaR), Capan‐1TalR (TalR). Error bars indicate standard deviation (*n* = 3). *p < 0.05; **p < 0.005.

RNA sequencing found no meaningful differences in PARP1 or ABCG2 expression in Capan‐1OlaR and TalR cells compared to parental cells, while ABCB1 was not expressed in any of the cell lines which corresponds with the lack of ABCB1 mRNA and protein expression in the Human Protein Atlas [31]. We also analysed the expression levels of PARP1 and ABCG2 using Western blotting (Figure 3). The expression of PARP1 was significantly decreased by 34% in Capan‐1TalR cells (*p* = 0.005) compared to parental cells, whereas PARP1 expression in Capan‐1OlaR remained largely unchanged (*p* = 0.65). Surprisingly, significant downregulation of ABCG2 protein was observed in both resistant cell lines; in Capan‐1OlaR, expression diminished by 41% (*p* = 0.03), and in Capan‐1TalR, expression diminished by 71% (*p* = 0.003).

### 3.4 Olaparib and Talazoparib Induce Differential Alterations in Metabolomic Profile

**FIGURE 4:**
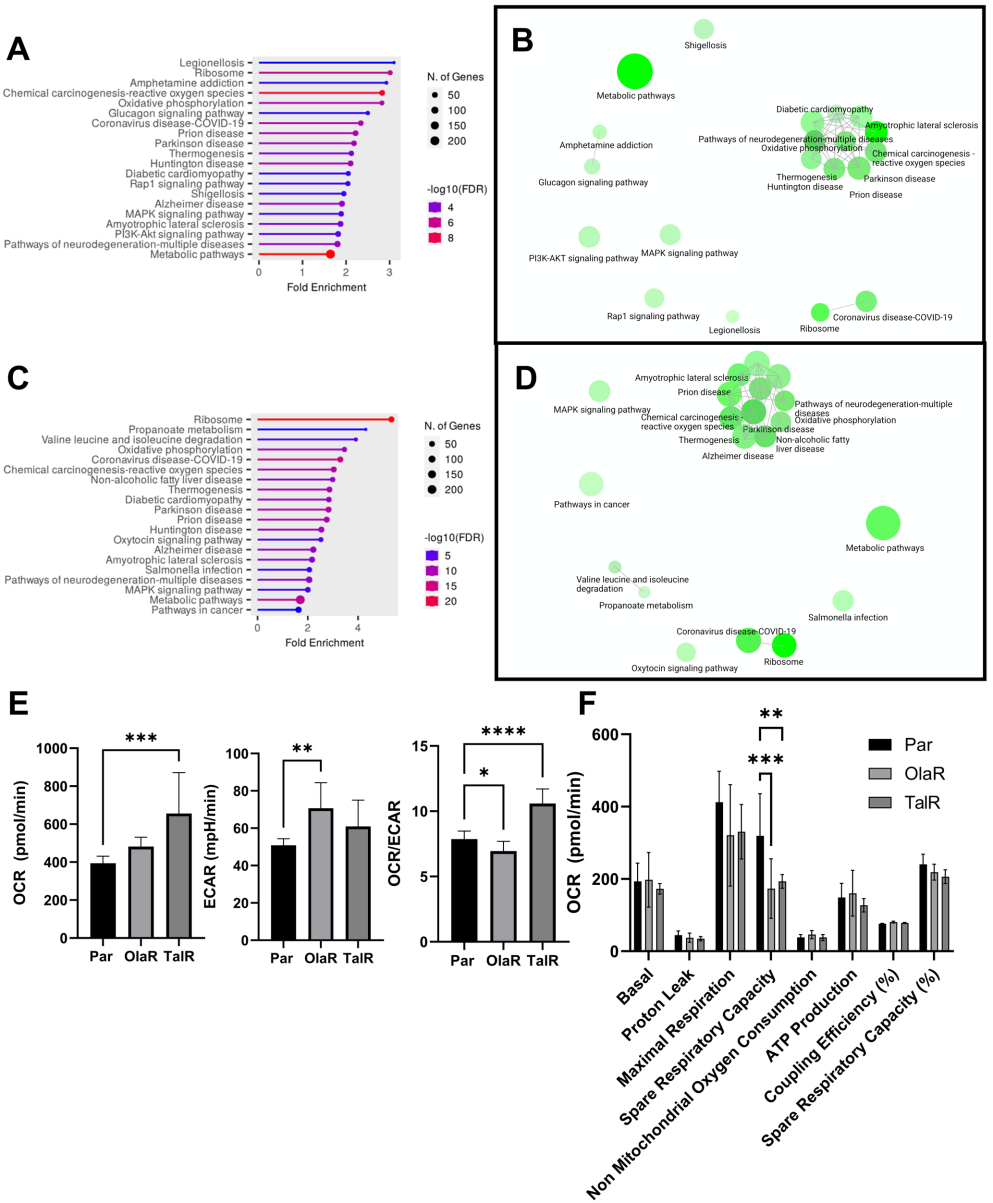
RNA sequencing and metabolomic profiling identify metabolomic alterations in olaparib‐ and talazoparib‐resistant Capan‐1 cells. (A–D) KEGG pathway analysis and clustering by ShinyGO for Capan‐1OlaR (A, B) and Capan‐1TalR (C, D). The size of each node indicates the number of genes in each pathway. The darker the node, the more significantly enriched the pathway is. Nodes or pathways that share more than 20% of their genes are connected by an edge. (E) metabolomic profiling of oxygen consumption rate (OCR), extracellular acidification rate (ECAR) and OCR/ECAR ratio under baseline conditions by Seahorse ATP rate assay. (F) OCR by various processes as measured by Seahorse MitoStress Test. Stars indicate statistical significance based on one‐way ANOVA followed by Dunnett's multiple comparisons test with parental cells set as the control (*p < 0.05; **p < 0.01; ***p < 0.001; ****p < 0.0001) (*n* = 3).

To elucidate novel resistance mechanisms to PARPi we performed differential expression and pathway analysis in parental and PARPi‐resistant Capan‐1. Compared to parental cells, we found 523 upregulated and 1506 downregulated genes in Capan‐1OlaR, and 740 upregulated and 1241 downregulated genes in Capan‐1TalR (probability > 0.9) (Figure S1). Subsequently, pathway analysis showed that differentially expressed genes were associated with several pathways linked to metabolism, including the main cluster ‘metabolic pathways’, as well as ‘oxidative phosphorylation’ and ‘chemical carcinogenesis—reactive oxygen species’ (Figure 4A–D). We therefore further investigated cellular metabolism by Seahorse assay. Compared to parental cells, Capan‐1OlaR had a significantly higher extracellular acidification rate (ECAR, *p* = 0.006) whereas Capan‐1TalR had a significantly higher oxygen consumption rate (OCR, *p* = 0.0.001) (Figure 4E). When plotted as the OCR:ECAR ratio, Capan‐1OlaR had a significant decrease in ratio (−9.3%, *p* = 0.04) indicative of a shift toward glycolysis and Capan‐1TalR had a significant increase in ratio (27.4%, *p* < 0.0001) indicative of a shift toward oxidative phosphorylation (OxPhos). Interestingly, both resistant lines had a significantly lower spare respiratory capacity than the parental cells (Par: 319.5 pmol/min, OlaR: 173.5 pmol/min and *p* = 0.0001, TalR: 193.0 pmol/min and *p* = 0.003) (Figure 4F).

**FIGURE 5:**
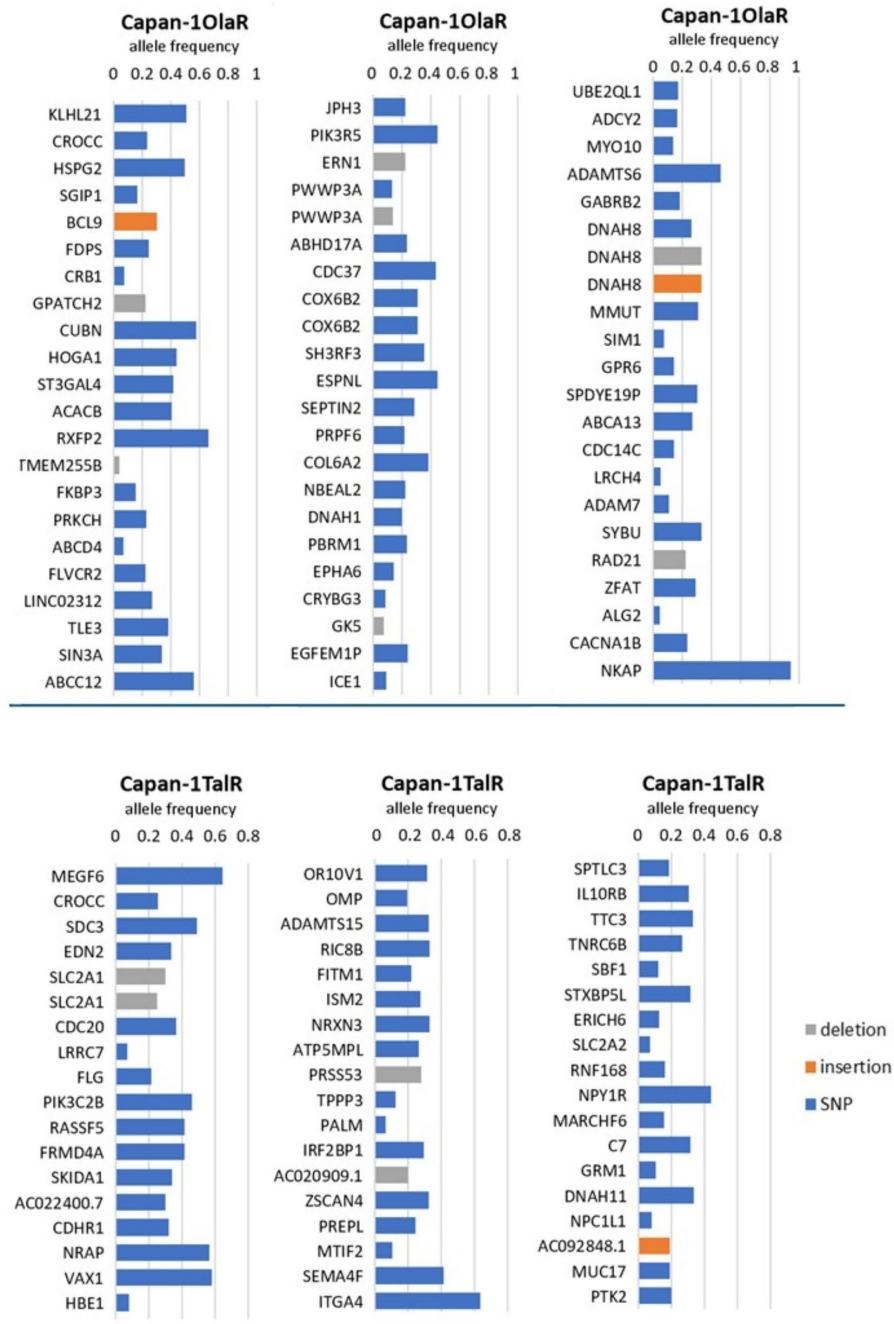
Allele frequencies of mutated genes in Capan‐1 resistant cell lines. The genome of the Capan‐1 parental cells was set as control to select alterations specific to the resistant cells. Blue: Single nucleotide variant; orange: Insertion; grey: Deletion (*n* = 1).

We evaluated the protein expression level of cytochrome c oxidase subunit 4 (Cox4) and glycogen synthase kinase 3 (GSK3β) as regulators of oxidative phosphorylation and glycolysis, respectively. No significant differences were observed in the protein expression of either protein (Figure S2). Whole exome sequencing found 62 novel alterations in Capan‐1OlaR and 53 novel alterations in Capan‐1TalR compared to the parental cells (Figure 5). The majority of the alterations were single nucleotide variants, although several insertions and deletions were also found. In Capan‐1OlaR, mutations were found in six genes involved in metabolism (*HOGA1, ACACB, ERN1, COX6B2, SEPTIN2, ABCA13*). In Capan‐1TalR, mutations were found in four genes involved in metabolism (*SLC2A1, FITM1, ATP5MPL* and *PRSS53*).

### 3.5 Talazoparib Induces a Similar Resistance Profile as Cisplatin

**FIGURE 6:**
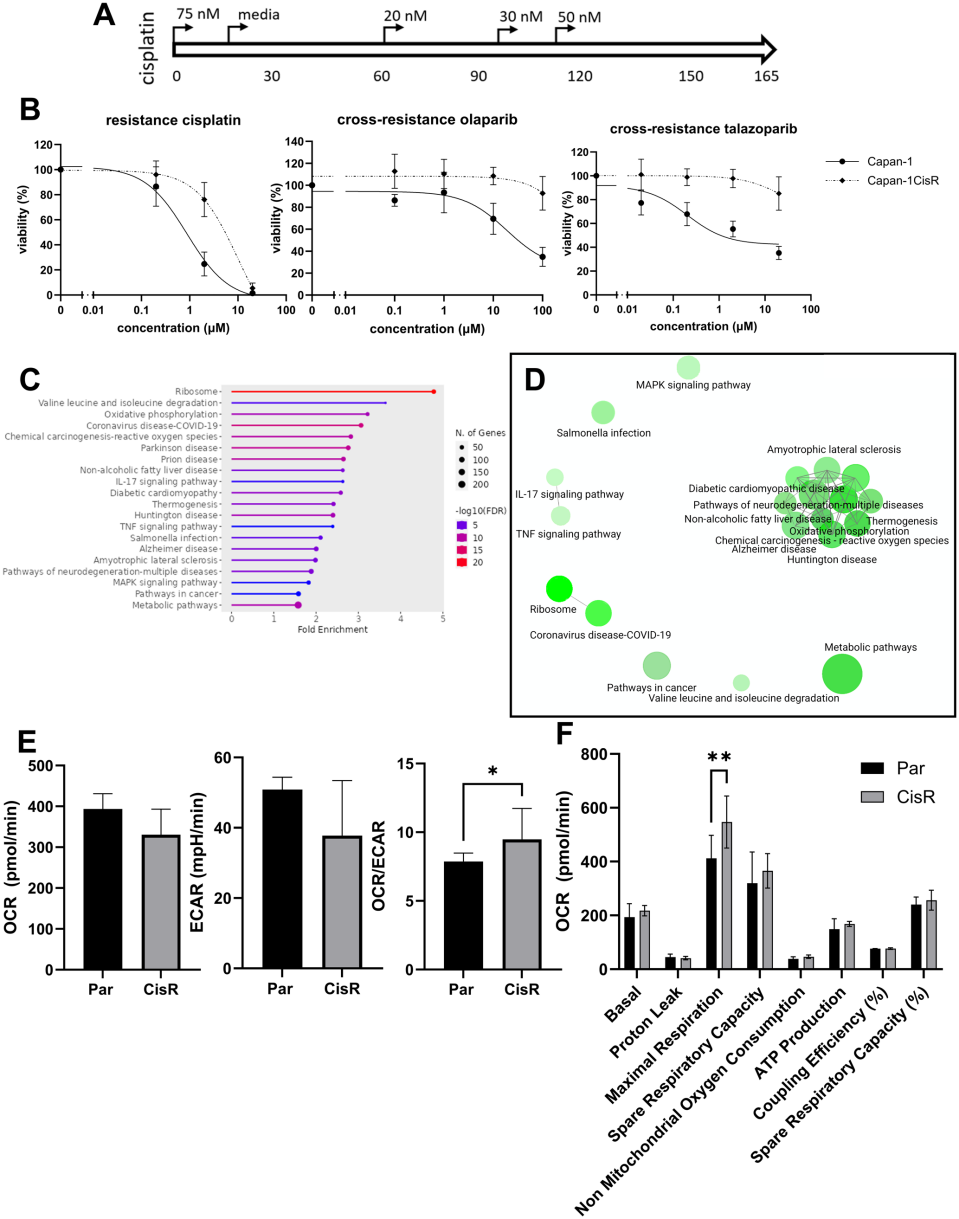
Development and characterisation of cisplatin‐resistant Capan‐1 cell line Capan‐1CisR. (A) Treatment schedule. (B) Viability assay in parental and resistant cells after treatment with cisplatin, olaparib, or talazoparib for 7 days as measured by PrestoBlue staining. (C, D) KEGG pathway analysis and clustering by ShinyGO for Capan‐1CisR. The size of each node indicates the number of genes in each pathway. The darker the node, the more significantly enriched the pathway is. Nodes or pathways that share more than 20% of their genes are connected by an edge. (E) metabolomic profiling of oxygen consumption rate (OCR), extracellular acidification rate (ECAR) and OCR/ECAR ratio under baseline conditions by Seahorse ATP rate assay. (F) OCR by various processes as measured by Seahorse MitoStress Test. Stars indicate statistical significance based on one‐way ANOVA followed by Dunnett's multiple comparisons test with parental cells set as the control (*p < 0.05; **p < 0.01) (*n* = 3).

Talazoparib has a higher PARP1 trapping potential compared to olaparib, which may contribute to a more genotoxic effect. Since Capan‐1OlaR and Capan‐1TalR showed different mechanisms of resistance, we also developed a cisplatin‐resistant Capan‐1 cell line (Capan‐1CisR) to investigate if there are similarities between the mechanisms of resistance to PARPi and platinum agents (Figure 6A).

The IC_50_ value of cisplatin‐resistant Capan‐1 cells increased 6.6‐fold compared to parental cells (5.3 vs. 0.8 μM) (Figure 6B). Capan‐1CisR also displayed significant cross‐resistance to olaparib, with a viability of 93% at 100 μM compared to 35% in the parental cells (*p* < 0.002), and talazoparib, with a viability of 85% at 20 μM compared to 35% in the parental cells (*p* < 0.005). Additionally, Capan‐1CisR displayed significant cross‐resistance to oxaliplatin but showed no significant cross‐resistance to gemcitabine, 5‐FU, or FOLFIRINOX (Table S1 and Figure S3).

Similar to Capan‐1TalR, protein levels of PARP1 and ABCG2 were significantly decreased in Capan‐1CisR (Figure S4). RNA sequencing and KEGG pathway analysis identified enrichment of the same metabolism‐associated pathways as Capan‐1OlaR and TalR (‘metabolic pathways’, ‘oxidative phosphorylation’ and ‘chemical carcinogenesis—reactive oxygen species’). We therefore also performed in vitro metabolomic profiling of Capan‐1CisR and observed a significant increase in OCR/ECAR ratio (*p* = 0.02), as well as a significant increase in maximal respiration (*p* = 0.002) (Figure 6E,F).

## 4 Discussion

Homologous recombination deficiency due to BRCA1/2 or PALB2 mutations is known to sensitise to PARP inhibitors. Olaparib is one of the few agents that have improved progression‐free survival of BRCAmut PDAC patients when used as maintenance treatment after disease control with first‐line platinum agent [32, 33]. However, rapid acquisition of resistance is an issue and olaparib did not demonstrate an improvement in overall survival in this setting. Other novel PARPi have shown increased efficacy over olaparib in other HRD solid tumours and have potential in PDAC as well [4, 34, 35]. Nevertheless, acquisition of resistance as well as cross‐resistance remains a concern. In this study, we developed and characterised BRCA2‐mutant olaparib‐ and talazoparib‐resistant Capan‐1 cells. Both cell lines showed significant cross‐resistance to the other PARPi and to oxaliplatin. Additionally, Capan‐1TalR, but not Capan‐1OlaR, showed significant cross‐resistance to cisplatin and FOLFIRINOX. Cross‐resistance between PARPi and platinum agents has been described in multiple studies and may be attributed to overlapping mechanisms of resistance [36, 37, 38]. In line with this, we also observed significant cross‐resistance from Capan‐1CisR to both PARPi. The lack of cross‐resistance of Capan‐1OlaR to cisplatin suggests that these cells have a different mechanism of resistance compared to Capan‐1TalR.

Currently, the standard‐of‐care is to provide either FOLFIRINOX or gemcitabine plus nab‐paclitaxel as first‐line treatment for patients with unresectable disease, although gemcitabine plus cisplatin is also recommended for patients with gBRCA mutations [39, 40]. Olaparib is given to patients with BRCA mutation either as maintenance therapy when patients do not progress on first‐line therapy, or as second‐line therapy. After progression on olaparib, a modified version of FOLFIRINOX is commonly given as third‐line therapy. Our Capan‐1CisR cells show significant cross‐resistance to both PARPi, which might explain the limited survival benefit of olaparib treatment in patients who have progressed on cisplatin. Additionally, our Capan‐1OlaR cells show significant cross‐resistance to oxaliplatin and a decreased sensitivity to FOLFIRINOX but remain sensitive to gemcitabine and 5‐FU. We therefore suggest that gemcitabine (and to a lesser extent 5‐FU which is part of FOLFIRINOX) may be a more effective third‐line treatment than a modified form of FOLFIRINOX. Alternatively, two studies have found synergism between PARPi and gemcitabine in several PDAC cell lines, suggesting that the combination of olaparib and gemcitabine may improve treatment response [41, 42].

Previous studies have identified restoration of BRCA2 mutation as one of the main mechanisms of resistance. We observed no reversal mutations. Other previously described resistance mechanisms are loss of PARP1 expression and overexpression of drug efflux pump ABCG2. We found a significant decrease in PARP1 protein expression in Capan‐1TalR but not in Capan‐1OlaR. Loss of PARP1 expression has been associated with acquired resistance and is hypothesised to protect the cell from the formation of cytotoxic PARP‐DNA complexes [13, 43]. The mechanism of action of PARPi is two‐fold: inhibition of PARylation by binding to the NAD^+^ pocket of PARP1 and trapping of the PARP1 protein on the DNA. PARP‐trapping potency has been shown to strongly correlate with PARPi cytotoxicity [44]. The mechanism and effect of PARP trapping remain to be elucidated but the currently favoured hypothesis is that a combination of enzymatic inhibition and a change in PARP affinity for DNA results in PARP trapping which in turn blocks replication fork progression and causes double‐strand breaks (DSBs). However, recent studies have instead linked PARPi cytotoxic activity to inhibition of binding of other DDR proteins at DSBs [45] and to transcription‐replication conflicts caused by PARP inhibition rather than trapping [46]. Talazoparib has a 100‐fold higher trapping potency compared to olaparib which may be due to its more extensive interactions with the active sites of PARP1 [44]. Differences in inhibitor structure and PARP1‐interaction may affect their mechanism of action and contribute to differences in resistance mechanisms.

In contrast to previous observations [12, 47, 48], we observed significant loss of ABCG2 protein expression in all resistant Capan‐1 cells. The effect of downregulation on acquired cisplatin or PARPi resistance is unclear and warrants further investigation. Olaparib and talazoparib, but not cisplatin, are substrates of ABCG2 [12, 49, 50, 51]. In non‐small cell lung cancer, ABCG2 expression was found to be significantly lower in primary cancer tissues compared to healthy tissue [52]. Inhibition of beta‐catenin down‐regulated ABCG2 expression. Beta‐catenin is a major regulator of the Wnt pathway and is inhibited by non‐canonical Wnt5a. Treatment with cisplatin down‐regulated Wnt5a and significantly increased canonical Wnt7b expression, which increased ABCG2 levels through activation of the beta‐catenin pathway. In PDAC, Wnt5a is highly expressed, but in contrast to the previous study, this has been positively correlated with ABCG2 expression and gemcitabine resistance although no correlation has been found with survival [53, 54, 55]. Additionally, in breast tumours ABCG2 expression was not significantly altered in olaparib naïve or resistant tumours but was significantly different between adenocarcinoma and metaplastic spindle cell carcinoma, suggesting that ABCG2 expression is phenotype dependent [56]. We observed significant loss of beta‐catenin and ABCG2 expression in Capan‐1CisR and Capan‐1TalR (Figure S4) which suggests that modulation of the Wnt pathway may be involved in the development of resistance in our cells, although further research is required.

To identify novel mechanisms of resistance to PARPi, we performed transcriptomic profiling of the parental and resistant cells. KEGG pathway analysis found significant enrichment of the metabolomic and oxidative phosphorylation pathways in both resistant cell lines. Because both pathways are associated with energy production and metabolism, we expanded our investigation to in vitro metabolomic profiling using Seahorse assays. These assays measure the OCR and ECAR, which can be taken as measures of OxPhos and glycolysis, respectively.

We observed an increase in metabolism due to a significant increase in glycolysis (ECAR) in Capan‐1OlaR, which was reflected in a significant decrease in the OCR/ECAR ratio. An increase in metabolism was also observed in Capan‐1TalR, but in contrast to Capan‐1OlaR, this was due to a significant increase in OxPhos (OCR) which was also reflected in a significant increase in OCR/ECAR. The increase in metabolism compared to the parental cells is in accordance with the increased proliferation rate. We also observed a strong and significant decrease in spare respiratory capacity (SRC) in these cells. The SRC is the difference between the maximal respiratory capacity and the basal respiratory rate and indicates a cell's capacity to meet increased energetic demand in response to temporary energy taxing processes and is a marker for metabolic fitness [57]. Compared to normal cells, SRC is commonly lower in cancer cells and is considered a metabolic weakness, which has been further enhanced in our PARPi‐resistant cells.

We investigated protein expression of Cox4 and GSK3β to investigate changes in OxPhos and glycolysis, respectively, but did not find significant alterations in protein expression. Cox4 was selected for Western blot analysis because it is part of the cytochrome C complex and is therefore important for OxPhos and may provide information about mitochondrial function. However, this does mean that it is very far downstream in the OxPhos pathway and there are many regulatory proteins upstream whose changes in expression may have a stronger effect on pathway regulation [58]. We therefore also analysed protein expression of GSK3b, which is a well‐known negative regulator of glucose homeostasis, and changes in protein expression have been associated with cancer. The actual role of GSK3b is more complicated, however, as GSK3b is a central node in both the PI3K/AKT/mTOR, AMPK and Wnt signalling pathways and the effect of alterations in expression is likely context dependent as contradicting effects have been described [59, 60]. While a change in GSK3b expression would have been a clear indication of a metabolic shift, a lack of change does not necessarily indicate a lack of metabolic shift. Further research will include proteomic and metabolomic profiling to elucidate functional alterations in metabolic pathways and identify potential drug targets.

Since Capan‐1OlaR and Capan‐1TalR showed different mechanisms of resistance, we also developed a cisplatin‐resistant Capan‐1 cell line (Capan‐1CisR) to investigate if there are similarities between the mechanisms of resistance to PARPi and platinum agents. Unexpectedly, like Capan‐1TalR, Capan‐1CisR had a significant loss of PARP1 expression. PARP1 overexpression has previously been associated with cisplatin resistance [61] and may to some degree explain the observed cross‐resistance to olaparib and talazoparib. In contrast to both PARPi‐resistant lines, Capan‐1CisR showed a decrease in both OCR and ECAR, which reflects a decrease in general metabolism. While the changes in OCR and ECAR individually were not significant, the OCR/ECAR ratio showed a significant increase, suggesting that these cells (like Capan‐1TalR) are more dependent on OxPhos. The question remains why the two PARPi‐resistant cell lines have distinct metabolic alterations. Recently, Zampieri et al. found that olaparib, not talazoparib, is a mitochondrial complex I inhibitor [62]. Olaparib reduced mitochondrial OCR in a temozolomide‐resistant glioblastoma cell line, while talazoparib did not impact OCR. Inhibition of OCR by olaparib would promote upregulation of glycolysis, as we observed in Capan‐1OlaR.

Lahiguera et al. showed that HRD tumours are more dependent on oxidative phosphorylation and suggest that increased oxidative phosphorylation helps to maintain high ATP and NAD^+^ levels to ensure PARP‐dependent DNA repair [63]. As PARPi uses the same binding site at NAD^+^ [44], high NAD^+^ levels could also compete with PARPi binding and thereby reduce PARPi efficacy. On the other hand, Lahiguera et al. also showed that high glycolytic metabolism decreased the effect of olaparib similar to our observation in Capan‐1OlaR. While the mechanism is still unclear, they suggest that high glycolysis may reduce the capacity of olaparib to block PARP activity or alter the cell cycle.

Metabolism is a burgeoning field in PDAC research and while targeted therapies against metabolism are still a long way from routine treatment, there are numerous clinical trials running that investigate a variety of metabolomic pathways [64, 65]. One of the treatments that is receiving a considerable amount of attention is the metabolic regulator metformin [66]. Metformin has been shown to increase the production of reactive oxygen species and decrease the mitochondrial membrane potential through the release of cytochrome C and increased olaparib sensitivity in ovarian cancer cell lines [67]. Additionally, in a pan‐cancer cell line panel, metformin was found to have a positive correlation with SRC, suggesting that PARPi resistance could further enhance sensitivity to metformin [68]. Metformin has also been shown to sensitise to olaparib in ovarian cancer cell lines irrespective of BRCA status [67, 69], and inhibit PARPi‐mediated epithelial‐mesenchymal transition in PARPi‐resistant triple‐negative breast cancer cell lines [70]. Furthermore, several in vitro and in vivo studies as well as two clinical trials have shown synergism between cisplatin and metformin (reviewed in [71]).

Further investigation of the role and potential of targeting metabolism in PDAC is needed, and our resistant Capan‐1 cell lines provide valuable models to do this. Future work will include more in‐depth investigation of the role of metabolism in PARPi resistance by proteomic and metabolomic profiling and how metabolic inhibitors such as metformin and lonidamine can be used to prevent or overcome metabolism‐mediated PARPi resistance. Additionally, we will investigate whether the increased dependence on OxPhos, as observed in Capan‐1TalR and Capan‐1CisR, has similar causes and can be used to improve treatment.

In this study, we have developed PARP inhibitor and cisplatin‐resistant BRCA2 mutant Capan‐1 cell lines and have identified novel mechanisms of resistance. Our findings underscore the intricate nature of resistance mechanisms to PARP inhibitors in pancreatic ductal adenocarcinoma (PDAC) and point to potential metabolic therapeutic targets. The observed differences in the resistance profiles between olaparib and talazoparib suggest that the potency of PARP trapping may contribute to the development of resistance. Additional studies are required to confirm these results and to investigate innovative therapeutic approaches aimed at overcoming PARP inhibitor resistance in PDAC.

Numerous studies have investigated and identified resistance mechanisms to PARPi, with the most well‐known mechanisms being the restoration of BRCA1/2 function through reversal mutations and the upregulation of drug efflux pumps [9, 12, 13, 29, 30]. However, these alterations do not account for all cases with PARPi resistance in the clinic, and alternative mechanisms have also been described. In this study, we did not observe alterations in BRCA1/2 or other HR genes that were not already present in the parental Capan‐1 cells or overexpression of drug efflux pumps. However, we did observe significant downregulation of PARP1 expression as well as alterations in cellular metabolism. These findings, combined with the numerous alternative processes described in the literature, highlight the multi‐faceted nature of drug resistance. It is likely that resistance is rarely caused by a single genetic alteration but rather a combination of multiple processes that, on their own, have little to no impact on drug sensitivity. This also explains the difficulty of preventing, predicting, or overcoming resistance in the clinic. Repeated study of resistance mechanisms will allow us to gain a better understanding of the processes associated with resistance and bring us closer to improving treatment success in the clinic.

To conclude, acquired resistance to genotoxic and targeted therapies remains a large problem in improving cancer survival. Here we established and studied clinically relevant acquired resistance to PARPi olaparib and talazoparib, as well as the platinum agent cisplatin in the BRCA2 mutant PDAC cell line Capan‐1. We observed significant cross‐resistance to multiple commonly used therapeutic agents, but observed no significant cross‐resistance to gemcitabine and 5‐FU in any of the three resistant cell lines, highlighting the potential for follow‐up treatment after progression on PARPi or cisplatin. Evaluation of drug resistance mechanisms showed that drug resistance is a highly multi‐faceted process that involves multiple pathways. All three resistant cell lines had significant alterations in metabolism, including a reduction in spare respiratory capacity, which may provide an opportunity to target resistant cells. Since the cells had unique metabolic pathway alterations, further research is necessary to elucidate the exact mechanism of these alterations and how they can be targeted using metabolic inhibitors.

## Author Contributions

**Jojanneke Stoof:** conceptualization (equal), investigation (equal), visualization (equal), writing – original draft (equal), writing – review and editing (equal). **Charlotte Andrieu:** formal analysis (equal), writing – review and editing (equal). **Fiona O'Connell:** investigation (equal), writing – review and editing (equal). **Jacintha O'Sullivan:** conceptualization (equal), writing – review and editing (equal). **Maeve A. Lowery:** conceptualization (equal), funding acquisition (equal), supervision (equal), writing – review and editing (equal). **Naomi Walsh:** conceptualization (equal), funding acquisition (equal), writing – original draft (equal), writing – review and editing (equal).

## Ethics Statement

The authors have nothing to report.

## Consent

The authors have nothing to report.

## Conflicts of Interest

The authors declare no conflicts of interest.

## Supporting information


**Table S1:** IC_50_ values for commonly used targeted and chemotherapeutic agents in Capan‐1 parental and resistant cell lines.
**Figure S1:** Similarities in gene expression between Capan‐1 parental and resistant lines. Based on RNA sequencing data. (A) Multidimensional scaling plot. (B) Mean‐difference plot for Capan‐1CisR. (C) Mean‐difference plot for Capan‐1OlaR. (D) Mean‐difference plot for Capan‐1TalR (*n* = 1).
**Figure S2:** Protein expression of Cox4 and Gsk3beta as measured by Western blotting. The bands for beta‐actin are duplicated from Figure 3, with the addition of the previously excluded CisR sample.
**Figure S3:** Viability assay in parental and resistant cells after seven days of treatment as measured by PrestoBlue staining (*n* = 3).
**Figure S4:** Expression of beta‐catenin, PARP1 and ABCG2 in Capan‐1 by Western blot. This figure is an extended version of Figure 3 which includes the previously excluded CisR sample. Expression was quantified using ImageJ and corrected against the loading control beta‐actin and then normalised against the parental cells. Student's *t*‐test was used to identify statistical differences between each resistant cell line and the parental cell line. Error bars show the standard deviation of the mean (*n* = 3). *p < 0.05; **p < 0.005; ***p < 0.001.

## Data Availability

DNA and RNA sequencing data of this study have been deposited in the Sequence Read Archive (SRA) as fastq files under accession number PRJNA1263861 (https://www.ncbi.nlm.nih.gov/sra/PRJNA1263861).
