# Supplementary material for: Mechanisms of Resistance to PARPi in Pancreatic Ductal Adenocarcinoma
Source: J Cell Mol Med. 2025 Aug 28;29(16):e70816. doi: 10.1111/jcmm.70816 (PMC12392133; doi:10.1111/jcmm.70816)
Supplement: Supplementary file 1 — Table S1: IC50 values for commonly used targeted and chemotherapeutic agents in Capan‐1 parental and resistant cell lines. Figure S1: Similarities in gene expression between Capan‐1 parental and resistant lines. Based on RNA sequencing data. (A) Multidimensional scaling plot. (B) Mean‐difference plot for Capan‐1CisR. (C) Mean‐difference plot for Capan‐1OlaR. (D) Mean‐difference plot for Capan‐1TalR (n = 1). Figure S2: Protein expression of Cox4 and Gsk3beta as measured by Western blotting. The bands for beta‐actin are duplicated from Figure 3, with the addition of the previously excluded CisR sample. Figure S3: Viability assay in parental and resistant cells after seven days of treatment as measured by PrestoBlue staining (n = 3). Figure S4: Expression of beta‐catenin, PARP1 and ABCG2 in Capan‐1 by Western blot. This figure is an extended version of Figure 3 which includes the previously excluded CisR sample. Expression was quantified using ImageJ and corrected against the loading control beta‐actin and then normalised against the parental cells. Student's t‐test was used to identify statistical differences between each resistant cell line and the parental cell line. Error bars show the standard deviation of the mean (n = 3). *p < 0.05; **p < 0.005; ***p < 0.001. [file JCMM-29-e70816-s001.docx]

## 7. Supplementary data

***Supplemental table 1. IC_50_ values for commonly used targeted and chemotherapeutic agents in Capan-1 parental and resistant cell lines***

| **Drug** | **Reported C_max, plasma_** | | **IC_50_ (standard deviation, p-value)** | | | |
| --- | --- | --- | --- | --- | --- | --- |
|  |  |  | **Capan-1** | **Capan-1CisR** | **Capan-1OlaR** | **Capan-TalR** |
| Cisplatin | 30 μM | [[1]](https://web.endnote.com/citations/eyJkaXNwbGF5VGV4dCI6IlsxXSIsImNpdGF0aW9ucyI6W3siYmlibGlvQ29udGVudCI6W3siZ3JvdXBHdWlkcyI6W10sImF1dGhvcnMiOlsiUHJhZGVlcCBSYWprdW1hciIsIkJpbnUgU3VzYW4gTWF0aGV3IiwiU2Fpa2F0IERhcyIsIlJhamVzaCBJc2FpYWgiLCJTdWJhc2hpbmkgSm9obiIsIlJhdG5hIFByYWJoYSIsIkRlbmlzZSBIZWxlbiBGbGVtaW5nIl0sInZvbHVtZSI6IjEwIiwidGl0bGUiOiJDaXNwbGF0aW4gQ29uY2VudHJhdGlvbnMgaW4gTG9uZyBhbmQgU2hvcnQgRHVyYXRpb24gSW5mdXNpb246IEltcGxpY2F0aW9ucyBmb3IgdGhlIE9wdGltYWwgVGltZSBvZiBSYWRpYXRpb24gRGVsaXZlcnkiLCJ1cmwiOlsiaHR0cHM6Ly93d3cubmNiaS5ubG0ubmloLmdvdi9wbWMvYXJ0aWNsZXMvUE1DNTAyMDE5NC8iXSwibnVtYmVyIjoiNyIsInNlY29uZGFyeVRpdGxlIjoiSm91cm5hbCBvZiBDbGluaWNhbCBhbmQgRGlhZ25vc3RpYyBSZXNlYXJjaCA6IEpDRFIiLCJyZWZlcmVuY2VUeXBlIjoiMTciLCJyc3htbCI6IjxyZWNvcmQ%2BPHJlZi10eXBlPjE3PC9yZWYtdHlwZT48Y29udHJpYnV0b3JzPjxhdXRob3JzPjxhdXRob3I%2BPHN0eWxlIHNpemU9XCIxMDAlXCIgZm9udD1cImRlZmF1bHRcIj5QcmFkZWVwIFJhamt1bWFyPC9zdHlsZT48L2F1dGhvcj48YXV0aG9yPjxzdHlsZSBzaXplPVwiMTAwJVwiIGZvbnQ9XCJkZWZhdWx0XCI%2BQmludSBTdXNhbiBNYXRoZXc8L3N0eWxlPjwvYXV0aG9yPjxhdXRob3I%2BPHN0eWxlIHNpemU9XCIxMDAlXCIgZm9udD1cImRlZmF1bHRcIj5TYWlrYXQgRGFzPC9zdHlsZT48L2F1dGhvcj48YXV0aG9yPjxzdHlsZSBzaXplPVwiMTAwJVwiIGZvbnQ9XCJkZWZhdWx0XCI%2BUmFqZXNoIElzYWlhaDwvc3R5bGU%2BPC9hdXRob3I%2BPGF1dGhvcj48c3R5bGUgc2l6ZT1cIjEwMCVcIiBmb250PVwiZGVmYXVsdFwiPlN1YmFzaGluaSBKb2huPC9zdHlsZT48L2F1dGhvcj48YXV0aG9yPjxzdHlsZSBzaXplPVwiMTAwJVwiIGZvbnQ9XCJkZWZhdWx0XCI%2BUmF0bmEgUHJhYmhhPC9zdHlsZT48L2F1dGhvcj48YXV0aG9yPjxzdHlsZSBzaXplPVwiMTAwJVwiIGZvbnQ9XCJkZWZhdWx0XCI%2BRGVuaXNlIEhlbGVuIEZsZW1pbmc8L3N0eWxlPjwvYXV0aG9yPjwvYXV0aG9ycz48L2NvbnRyaWJ1dG9ycz48dGl0bGVzPjx0aXRsZT48c3R5bGUgc2l6ZT1cIjEwMCVcIiBmb250PVwiZGVmYXVsdFwiPkNpc3BsYXRpbiBDb25jZW50cmF0aW9ucyBpbiBMb25nIGFuZCBTaG9ydCBEdXJhdGlvbiBJbmZ1c2lvbjogSW1wbGljYXRpb25zIGZvciB0aGUgT3B0aW1hbCBUaW1lIG9mIFJhZGlhdGlvbiBEZWxpdmVyeTwvc3R5bGU%2BPC90aXRsZT48c2Vjb25kYXJ5LXRpdGxlPjxzdHlsZSBzaXplPVwiMTAwJVwiIGZvbnQ9XCJkZWZhdWx0XCI%2BSm91cm5hbCBvZiBDbGluaWNhbCBhbmQgRGlhZ25vc3RpYyBSZXNlYXJjaCA6IEpDRFI8L3N0eWxlPjwvc2Vjb25kYXJ5LXRpdGxlPjwvdGl0bGVzPjxkYXRlcz48eWVhcj48c3R5bGUgc2l6ZT1cIjEwMCVcIiBmb250PVwiZGVmYXVsdFwiPjIwMTY8L3N0eWxlPjwveWVhcj48L2RhdGVzPjx2b2x1bWU%2BPHN0eWxlIHNpemU9XCIxMDAlXCIgZm9udD1cImRlZmF1bHRcIj4xMDwvc3R5bGU%2BPC92b2x1bWU%2BPGlzYm4%2BPHN0eWxlIHNpemU9XCIxMDAlXCIgZm9udD1cImRlZmF1bHRcIj4yMjQ5LTc4Mlg8L3N0eWxlPjwvaXNibj48YWJzdHJhY3Q%2BPHN0eWxlIHNpemU9XCIxMDAlXCIgZm9udD1cImRlZmF1bHRcIj5DaXNwbGF0aW4gaGFzIHJhZGlvc2Vuc2l0aXppbmcgcHJvcGVydGllcyBhbmQgdGhlIGJlc3Qgc2Vuc2l0aXphdGlvbiB0byByYWRpb3RoZXJhcHkgb2NjdXJzIHdpdGggYSBoaWdoZXIgcGxhc21hIGNvbmNlbnRyYXRpb24gb2YgY2lzcGxhdGluLiBUbyBvdXIga25vd2xlZGdlIHRoZSBvcHRpbWFsIHRpbWUgc2VxdWVuY2UgYmV0d2VlbiBjaGVtb3RoZXJhcHkgYW5kIGFkbWluaXN0cmF0aW9uIG9mIHJhZGlhdGlvbiB0aGVyYXB5LCB0byBvYnRhaW4gLi4uPC9zdHlsZT48L2Fic3RyYWN0Pjx1cmxzPjxyZWxhdGVkLXVybHM%2BPHVybD48c3R5bGUgc2l6ZT1cIjEwMCVcIiBmb250PVwiZGVmYXVsdFwiPmh0dHBzOi8vd3d3Lm5jYmkubmxtLm5paC5nb3YvcG1jL2FydGljbGVzL1BNQzUwMjAxOTQvPC9zdHlsZT48L3VybD48L3JlbGF0ZWQtdXJscz48L3VybHM%2BPGVsZWN0cm9uaWMtcmVzb3VyY2UtbnVtPjxzdHlsZSBzaXplPVwiMTAwJVwiIGZvbnQ9XCJkZWZhdWx0XCI%2BMTAuNzg2MC9KQ0RSLzIwMTYvMTgxODEuODEyNjwvc3R5bGU%2BPC9lbGVjdHJvbmljLXJlc291cmNlLW51bT48bnVtYmVyPjxzdHlsZSBzaXplPVwiMTAwJVwiIGZvbnQ9XCJkZWZhdWx0XCI%2BNzwvc3R5bGU%2BPC9udW1iZXI%2BPHJlYy1ndWlkPjdlNWQ4ZDIzLWRmZWUtNDY3OC1iNzAyLTQyODI4MTk4OTI4ZTwvcmVjLWd1aWQ%2BPHJlYy11c24%2BMzg5PC9yZWMtdXNuPjwvcmVjb3JkPiIsInJlY29yZFN0YXR1cyI6ImFjdGl2ZSIsInllYXIiOiIyMDE2IiwiZ3VpZCI6IjdlNWQ4ZDIzLWRmZWUtNDY3OC1iNzAyLTQyODI4MTk4OTI4ZSIsImVsZWN0cm9uaWNSZXNvdXJjZU51bWJlciI6IjEwLjc4NjAvSkNEUi8yMDE2LzE4MTgxLjgxMjYiLCJpc2JuIjoiMjI0OS03ODJYIn1dLCJyZWNvcmQiOnsiaXNibiI6IjIyNDktNzgyWCIsImVsZWN0cm9uaWMtcmVzb3VyY2UtbnVtIjoiMTAuNzg2MC9KQ0RSLzIwMTYvMTgxODEuODEyNiIsInRpdGxlcyI6eyJ0aXRsZSI6IkNpc3BsYXRpbiBDb25jZW50cmF0aW9ucyBpbiBMb25nIGFuZCBTaG9ydCBEdXJhdGlvbiBJbmZ1c2lvbjogSW1wbGljYXRpb25zIGZvciB0aGUgT3B0aW1hbCBUaW1lIG9mIFJhZGlhdGlvbiBEZWxpdmVyeSIsInNlY29uZGFyeS10aXRsZSI6IkpvdXJuYWwgb2YgQ2xpbmljYWwgYW5kIERpYWdub3N0aWMgUmVzZWFyY2ggOiBKQ0RSIn0sInJlYy1ndWlkIjoiN2U1ZDhkMjMtZGZlZS00Njc4LWI3MDItNDI4MjgxOTg5MjhlIiwiYWJzdHJhY3QiOiJDaXNwbGF0aW4gaGFzIHJhZGlvc2Vuc2l0aXppbmcgcHJvcGVydGllcyBhbmQgdGhlIGJlc3Qgc2Vuc2l0aXphdGlvbiB0byByYWRpb3RoZXJhcHkgb2NjdXJzIHdpdGggYSBoaWdoZXIgcGxhc21hIGNvbmNlbnRyYXRpb24gb2YgY2lzcGxhdGluLiBUbyBvdXIga25vd2xlZGdlIHRoZSBvcHRpbWFsIHRpbWUgc2VxdWVuY2UgYmV0d2VlbiBjaGVtb3RoZXJhcHkgYW5kIGFkbWluaXN0cmF0aW9uIG9mIHJhZGlhdGlvbiB0aGVyYXB5LCB0byBvYnRhaW4gLi4uIiwibnVtYmVyIjoiNyIsInJlZi10eXBlIjoiMTciLCJkYXRlcyI6eyJ5ZWFyIjoiMjAxNiJ9LCJyZWMtdXNuIjoiMzg5IiwidXJscyI6eyJyZWxhdGVkLXVybHMiOnsidXJsIjoiaHR0cHM6Ly93d3cubmNiaS5ubG0ubmloLmdvdi9wbWMvYXJ0aWNsZXMvUE1DNTAyMDE5NC8ifX0sImNvbnRyaWJ1dG9ycyI6eyJhdXRob3JzIjp7ImF1dGhvciI6WyJQcmFkZWVwIFJhamt1bWFyIiwiQmludSBTdXNhbiBNYXRoZXciLCJTYWlrYXQgRGFzIiwiUmFqZXNoIElzYWlhaCIsIlN1YmFzaGluaSBKb2huIiwiUmF0bmEgUHJhYmhhIiwiRGVuaXNlIEhlbGVuIEZsZW1pbmciXX19LCJ2b2x1bWUiOiIxMCJ9LCJndWlkIjoiN2U1ZDhkMjMtZGZlZS00Njc4LWI3MDItNDI4MjgxOTg5MjhlIn1dfQ%3D%3D) | 0.8 μM  (0.38) | 5.3 μM†  (3.6, 0.005) | 1.3 μM  (0.11, 0.1) | 6.6 μM†  (4.6, 0.002) |
| Olaparib | 30.1 μM | [[2, 3]](https://web.endnote.com/citations/eyJkaXNwbGF5VGV4dCI6IlsyLCAzXSIsImNpdGF0aW9ucyI6W3siYmlibGlvQ29udGVudCI6W3sicGxhY2VQdWJsaXNoZWQiOiJTw7ZkZXJ0w6RsamUiLCJ0aXRsZSI6IkNITVAgYXNzZXNzbWVudCByZXBvcnQgTHlucGFyemEiLCJncm91cEd1aWRzIjpbXSwicGFnZXMiOiIxODctMTg3IiwicmVjb3JkU3RhdHVzIjoiYWN0aXZlIiwiYXV0aG9ycyI6WyJBc3RyYVplbmVjYSwgQS4gQi4iXSwicmVmZXJlbmNlVHlwZSI6IjI3IiwieWVhciI6IjIwMTQiLCJndWlkIjoiMDdhM2JmNWMtMDFjYi00MmY5LWIzMGQtMzczNzRhZTc2NzQ2IiwicnN4bWwiOiI8cmVjb3JkPjxyZWYtdHlwZT4yNzwvcmVmLXR5cGU%2BPGNvbnRyaWJ1dG9ycz48YXV0aG9ycz48YXV0aG9yPjxzdHlsZSBzaXplPVwiMTAwJVwiIGZvbnQ9XCJkZWZhdWx0XCI%2BQXN0cmFaZW5lY2EsIEEuIEIuPC9zdHlsZT48L2F1dGhvcj48L2F1dGhvcnM%2BPC9jb250cmlidXRvcnM%2BPHRpdGxlcz48dGl0bGU%2BPHN0eWxlIHNpemU9XCIxMDAlXCIgZm9udD1cImRlZmF1bHRcIj5DSE1QIGFzc2Vzc21lbnQgcmVwb3J0IEx5bnBhcnphPC9zdHlsZT48L3RpdGxlPjwvdGl0bGVzPjxkYXRlcz48eWVhcj48c3R5bGUgc2l6ZT1cIjEwMCVcIiBmb250PVwiZGVmYXVsdFwiPjIwMTQ8L3N0eWxlPjwveWVhcj48L2RhdGVzPjxwYWdlcz48c3R5bGUgc2l6ZT1cIjEwMCVcIiBmb250PVwiZGVmYXVsdFwiPjE4Ny0xODc8L3N0eWxlPjwvcGFnZXM%2BPHB1Yi1sb2NhdGlvbj48c3R5bGUgc2l6ZT1cIjEwMCVcIiBmb250PVwiZGVmYXVsdFwiPlPDtmRlcnTDpGxqZTwvc3R5bGU%2BPC9wdWItbG9jYXRpb24%2BPHJlYy1ndWlkPjA3YTNiZjVjLTAxY2ItNDJmOS1iMzBkLTM3Mzc0YWU3Njc0NjwvcmVjLWd1aWQ%2BPHJlYy11c24%2BMjg8L3JlYy11c24%2BPC9yZWNvcmQ%2BIn1dLCJyZWNvcmQiOnsicmVjLWd1aWQiOiIwN2EzYmY1Yy0wMWNiLTQyZjktYjMwZC0zNzM3NGFlNzY3NDYiLCJwdWItbG9jYXRpb24iOiJTw7ZkZXJ0w6RsamUiLCJwYWdlcyI6IjE4Ny0xODciLCJjb250cmlidXRvcnMiOnsiYXV0aG9ycyI6eyJhdXRob3IiOiJBc3RyYVplbmVjYSwgQS4gQi4ifX0sInRpdGxlcyI6eyJ0aXRsZSI6IkNITVAgYXNzZXNzbWVudCByZXBvcnQgTHlucGFyemEifSwicmVjLXVzbiI6IjI4IiwiZGF0ZXMiOnsieWVhciI6IjIwMTQifSwicmVmLXR5cGUiOiIyNyJ9LCJndWlkIjoiMDdhM2JmNWMtMDFjYi00MmY5LWIzMGQtMzczNzRhZTc2NzQ2In0seyJyZWNvcmQiOnsicGFnZXMiOiIyMzMtMjMzIiwiY29udHJpYnV0b3JzIjp7ImF1dGhvcnMiOnsiYXV0aG9yIjoiQXN0cmFaZW5lY2EsIEEuIEIuIn19LCJkYXRlcyI6eyJ5ZWFyIjoiMjAxNyJ9LCJyZWMtZ3VpZCI6IjQ3MWU3ZTQzLTdiZTktNDYwZi04M2NhLTY2ZWU0YmQ0NmE4OCIsInVybHMiOnsicmVsYXRlZC11cmxzIjp7InVybCI6Imh0dHBzOi8vd3d3LmFjY2Vzc2RhdGEuZmRhLmdvdi9kcnVnc2F0ZmRhX2RvY3MvbmRhLzIwMTcvMjA4NTU4T3JpZzFzMDAwTXVsdGlkaXNjaXBsaW5lUi5wZGYifX0sInRpdGxlcyI6eyJ0aXRsZSI6Ik5EQS9CTEEgTXVsdGktZGlzY2lwbGluYXJ5IFJldmlldyBhbmQgRXZhbHVhdGlvbiBOREEgMjA4NTU4IEx5bnBhcnphVE0gKE9sYXBhcmliKSJ9LCJyZWYtdHlwZSI6IjI3IiwicmVjLXVzbiI6IjI5IiwicHViLWxvY2F0aW9uIjoiU8O2ZGVydMOkbGplIn0sImJpYmxpb0NvbnRlbnQiOlt7Imdyb3VwR3VpZHMiOltdLCJyZWNvcmRTdGF0dXMiOiJhY3RpdmUiLCJhdXRob3JzIjpbIkFzdHJhWmVuZWNhLCBBLiBCLiJdLCJ0aXRsZSI6Ik5EQS9CTEEgTXVsdGktZGlzY2lwbGluYXJ5IFJldmlldyBhbmQgRXZhbHVhdGlvbiBOREEgMjA4NTU4IEx5bnBhcnphVE0gKE9sYXBhcmliKSIsInllYXIiOiIyMDE3IiwiZ3VpZCI6IjQ3MWU3ZTQzLTdiZTktNDYwZi04M2NhLTY2ZWU0YmQ0NmE4OCIsInJzeG1sIjoiPHJlY29yZD48cmVmLXR5cGU%2BMjc8L3JlZi10eXBlPjxjb250cmlidXRvcnM%2BPGF1dGhvcnM%2BPGF1dGhvcj48c3R5bGUgc2l6ZT1cIjEwMCVcIiBmb250PVwiZGVmYXVsdFwiPkFzdHJhWmVuZWNhLCBBLiBCLjwvc3R5bGU%2BPC9hdXRob3I%2BPC9hdXRob3JzPjwvY29udHJpYnV0b3JzPjx0aXRsZXM%2BPHRpdGxlPjxzdHlsZSBzaXplPVwiMTAwJVwiIGZvbnQ9XCJkZWZhdWx0XCI%2BTkRBL0JMQSBNdWx0aS1kaXNjaXBsaW5hcnkgUmV2aWV3IGFuZCBFdmFsdWF0aW9uIE5EQSAyMDg1NTggTHlucGFyemFUTSAoT2xhcGFyaWIpPC9zdHlsZT48L3RpdGxlPjwvdGl0bGVzPjxkYXRlcz48eWVhcj48c3R5bGUgc2l6ZT1cIjEwMCVcIiBmb250PVwiZGVmYXVsdFwiPjIwMTc8L3N0eWxlPjwveWVhcj48L2RhdGVzPjxwYWdlcz48c3R5bGUgc2l6ZT1cIjEwMCVcIiBmb250PVwiZGVmYXVsdFwiPjIzMy0yMzM8L3N0eWxlPjwvcGFnZXM%2BPHB1Yi1sb2NhdGlvbj48c3R5bGUgc2l6ZT1cIjEwMCVcIiBmb250PVwiZGVmYXVsdFwiPlPDtmRlcnTDpGxqZTwvc3R5bGU%2BPC9wdWItbG9jYXRpb24%2BPHVybHM%2BPHJlbGF0ZWQtdXJscz48dXJsPjxzdHlsZSBzaXplPVwiMTAwJVwiIGZvbnQ9XCJkZWZhdWx0XCI%2BaHR0cHM6Ly93d3cuYWNjZXNzZGF0YS5mZGEuZ292L2RydWdzYXRmZGFfZG9jcy9uZGEvMjAxNy8yMDg1NThPcmlnMXMwMDBNdWx0aWRpc2NpcGxpbmVSLnBkZjwvc3R5bGU%2BPC91cmw%2BPC9yZWxhdGVkLXVybHM%2BPC91cmxzPjxyZWMtZ3VpZD40NzFlN2U0My03YmU5LTQ2MGYtODNjYS02NmVlNGJkNDZhODg8L3JlYy1ndWlkPjxyZWMtdXNuPjI5PC9yZWMtdXNuPjwvcmVjb3JkPiIsInBsYWNlUHVibGlzaGVkIjoiU8O2ZGVydMOkbGplIiwidXJsIjpbImh0dHBzOi8vd3d3LmFjY2Vzc2RhdGEuZmRhLmdvdi9kcnVnc2F0ZmRhX2RvY3MvbmRhLzIwMTcvMjA4NTU4T3JpZzFzMDAwTXVsdGlkaXNjaXBsaW5lUi5wZGYiXSwicmVmZXJlbmNlVHlwZSI6IjI3IiwicGFnZXMiOiIyMzMtMjMzIn1dLCJndWlkIjoiNDcxZTdlNDMtN2JlOS00NjBmLTgzY2EtNjZlZTRiZDQ2YTg4In1dfQ%3D%3D) | 32 μM  (22.3) | >100 μM | >100 μM | >100 μM |
| Talazoparib | 28.9-86.3 nM | [[4-6]](https://web.endnote.com/citations/eyJkaXNwbGF5VGV4dCI6Ils0LTZdIiwiY2l0YXRpb25zIjpbeyJiaWJsaW9Db250ZW50IjpbeyJ1cmwiOlsiaHR0cHM6Ly9saW5rLnNwcmluZ2VyLmNvbS8xMC4xMDA3L3MxMDYzNy0wMjMtMDEzNTEtdyJdLCJ5ZWFyIjoiMjAyMyIsInJlZmVyZW5jZVR5cGUiOiIxNyIsIm51bWJlciI6IjMiLCJzZWNvbmRhcnlUaXRsZSI6IkludmVzdGlnYXRpb25hbCBOZXcgRHJ1Z3MiLCJyc3htbCI6IjxyZWNvcmQ%2BPHJlZi10eXBlPjE3PC9yZWYtdHlwZT48Y29udHJpYnV0b3JzPjxhdXRob3JzPjxhdXRob3I%2BPHN0eWxlIHNpemU9XCIxMDAlXCIgZm9udD1cImRlZmF1bHRcIj5MdW8sIFlhbmc8L3N0eWxlPjwvYXV0aG9yPjxhdXRob3I%2BPHN0eWxlIHNpemU9XCIxMDAlXCIgZm9udD1cImRlZmF1bHRcIj5DaGVuZywgWWluZzwvc3R5bGU%2BPC9hdXRob3I%2BPGF1dGhvcj48c3R5bGUgc2l6ZT1cIjEwMCVcIiBmb250PVwiZGVmYXVsdFwiPld1LCBDaHVuamlhbzwvc3R5bGU%2BPC9hdXRob3I%2BPGF1dGhvcj48c3R5bGUgc2l6ZT1cIjEwMCVcIiBmb250PVwiZGVmYXVsdFwiPlllLCBIdWk8L3N0eWxlPjwvYXV0aG9yPjxhdXRob3I%2BPHN0eWxlIHNpemU9XCIxMDAlXCIgZm9udD1cImRlZmF1bHRcIj5DaGVuLCBOYWloYW48L3N0eWxlPjwvYXV0aG9yPjxhdXRob3I%2BPHN0eWxlIHNpemU9XCIxMDAlXCIgZm9udD1cImRlZmF1bHRcIj5aaGFuZywgRmFuPC9zdHlsZT48L2F1dGhvcj48YXV0aG9yPjxzdHlsZSBzaXplPVwiMTAwJVwiIGZvbnQ9XCJkZWZhdWx0XCI%2BV2VpLCBIdWE8L3N0eWxlPjwvYXV0aG9yPjxhdXRob3I%2BPHN0eWxlIHNpemU9XCIxMDAlXCIgZm9udD1cImRlZmF1bHRcIj5YdSwgQmluZ2hlPC9zdHlsZT48L2F1dGhvcj48L2F1dGhvcnM%2BPC9jb250cmlidXRvcnM%2BPHRpdGxlcz48dGl0bGU%2BPHN0eWxlIHNpemU9XCIxMDAlXCIgZm9udD1cImRlZmF1bHRcIj5QaGFybWFjb2tpbmV0aWNzLCBzYWZldHksIGFuZCBhbnRpdHVtb3IgYWN0aXZpdHkgb2YgdGFsYXpvcGFyaWIgbW9ub3RoZXJhcHkgaW4gQ2hpbmVzZSBwYXRpZW50cyB3aXRoIGFkdmFuY2VkIHNvbGlkIHR1bW9yczwvc3R5bGU%2BPC90aXRsZT48c2Vjb25kYXJ5LXRpdGxlPjxzdHlsZSBzaXplPVwiMTAwJVwiIGZvbnQ9XCJkZWZhdWx0XCI%2BSW52ZXN0aWdhdGlvbmFsIE5ldyBEcnVnczwvc3R5bGU%2BPC9zZWNvbmRhcnktdGl0bGU%2BPC90aXRsZXM%2BPGRhdGVzPjx5ZWFyPjxzdHlsZSBzaXplPVwiMTAwJVwiIGZvbnQ9XCJkZWZhdWx0XCI%2BMjAyMzwvc3R5bGU%2BPC95ZWFyPjwvZGF0ZXM%2BPHBhZ2VzPjxzdHlsZSBzaXplPVwiMTAwJVwiIGZvbnQ9XCJkZWZhdWx0XCI%2BNTAzLTUxMTwvc3R5bGU%2BPC9wYWdlcz48dm9sdW1lPjxzdHlsZSBzaXplPVwiMTAwJVwiIGZvbnQ9XCJkZWZhdWx0XCI%2BNDE8L3N0eWxlPjwvdm9sdW1lPjxhYnN0cmFjdD48c3R5bGUgc2l6ZT1cIjEwMCVcIiBmb250PVwiZGVmYXVsdFwiPlRhbGF6b3BhcmliLCBhIHBvbHkoQURQLXJpYm9zZSkgcG9seW1lcmFzZSBpbmhpYml0b3IsIGhhcyBkZW1vbnN0cmF0ZWQgZWZmaWNhY3kgaW4gdGhlIHRyZWF0bWVudCBvZiBhZHZhbmNlZCBicmVhc3QgYW5kIHByb3N0YXRlIGNhbmNlcnMgaW4gV2VzdGVybiBwb3B1bGF0aW9ucy4gVGhpcyBvcGVuLWxhYmVsLCBwaGFzZSAxIHN0dWR5IGludmVzdGlnYXRlZCB0aGUgcGhhcm1hY29raW5ldGljcywgc2FmZXR5LCBhbmQgYW50aXR1bW9yIGFjdGl2aXR5IG9mIHRhbGF6b3BhcmliIG1vbm90aGVyYXB5IGluIENoaW5lc2UgcGF0aWVudHMgd2l0aCBhZHZhbmNlZCBzb2xpZCB0dW1vcnMuIE1vbGVjdWxhcmx5IHVuc2VsZWN0ZWQgcGF0aWVudHMgKOKJpTE4IHllYXJzKSB3aXRoIGFkdmFuY2VkIHNvbGlkIHR1bW9ycyByZXNpc3RhbnQgdG8gc3RhbmRhcmQgdGhlcmFweSByZWNlaXZlZCB0YWxhem9wYXJpYiAob3JhbCwgMSBtZyBvbmNlIGRhaWx5KS4gUHJpbWFyeSBlbmRwb2ludCB3YXMgY2hhcmFjdGVyaXphdGlvbiBvZiBzaW5nbGUtZG9zZSBhbmQgc3RlYWR5LXN0YXRlIHBoYXJtYWNva2luZXRpY3MuIFNlY29uZGFyeSBlbmRwb2ludHMgZXZhbHVhdGVkIHNhZmV0eSwgdW5jb25maXJtZWQgb2JqZWN0aXZlIHJlc3BvbnNlIHJhdGUgKE9SUiksIGFuZCBkdXJhdGlvbiBvZiByZXNwb25zZS4gVGhlIHNhZmV0eSBwb3B1bGF0aW9uIGNvbXByaXNlZCAxNSBDaGluZXNlIHBhdGllbnRzIChtZWRpYW4gW3JhbmdlXSBhZ2UgNTMuMCBbMzEuMOKAkzcyLjBdIHllYXJzKS4gU2luZ2xlLWRvc2UgbWVkaWFuIHRpbWUgdG8gZmlyc3Qgb2NjdXJyZW5jZSBvZiBtYXhpbXVtIG9ic2VydmVkIGNvbmNlbnRyYXRpb24gd2FzIDEuOSBoOyBjb25jZW50cmF0aW9ucyB0aGVuIGRlY2xpbmVkIHdpdGggYSBtZWFuIHRlcm1pbmFsIGhhbGYtbGlmZSAodCAxLzIgKSBvZiA2NyBoLiBGb2xsb3dpbmcgbXVsdGlwbGUgZG9zaW5nLCBtZWRpYW4gVCBtYXggd2FzIGFwcHJveGltYXRlbHkgMS44NSBoIHdpdGggc3RlYWR5IHN0YXRlIGdlbmVyYWxseSBhY2hpZXZlZCBieSBEYXkgMjEuIFRyZWF0bWVudC1yZWxhdGVkIHRyZWF0bWVudC1lbWVyZ2VudCBhZHZlcnNlIGV2ZW50cyAoVEVBRXMpIG9jY3VycmVkIGluIDg2LjclICgxMy8xNSkgb2YgcGF0aWVudHMgKGdyYWRlIDMsIDIwLjAlOyBncmFkZSA0LCAxMy4zJSkuIFR3byBwYXRpZW50cyAoMTMuMyUpIGV4cGVyaWVuY2VkIHNlcmlvdXMgdHJlYXRtZW50LXJlbGF0ZWQgVEVBRXMuIE9SUiAoaW52ZXN0aWdhdG9yLWFzc2Vzc2VkKSB3YXMgNi43JSAoOTUlIENJOiAwLjLigJMzMS45KTsgb25lIHBhdGllbnQgKDYuNyUpIGhhZCBhIHBhcnRpYWwgcmVzcG9uc2UuIEluIHBhdGllbnRzIHdpdGggbWVhc3VyYWJsZSBkaXNlYXNlIGF0IGJhc2VsaW5lLCB0aGUgT1JSIHdhcyA5LjElICgxLzExOyA5NSUgQ0k6IDAuMuKAkzQxLjM7IGR1cmF0aW9uIG9mIHJlc3BvbnNlOiAxMTQgZGF5cyk7IHN0YWJsZSBkaXNlYXNlIHdhcyBhY2hpZXZlZCBieSAzNi40JSAoNC8xMSkgb2YgcGF0aWVudHMsIGFuZCA1NC41JSAoNi8xMSkgcHJvZ3Jlc3NlZCBieSBkYXRhIGN1dC1vZmYuIEluIENoaW5lc2UgcGF0aWVudHMgd2l0aCBhZHZhbmNlZCBzb2xpZCB0dW1vcnMsIHRoZSBwaGFybWFjb2tpbmV0aWMgcHJvZmlsZSBvZiB0YWxhem9wYXJpYiBtb25vdGhlcmFweSAoMSBtZy9kYXkpIHdhcyBjb25zaXN0ZW50IHdpdGggb3RoZXIgcGF0aWVudCBwb3B1bGF0aW9ucy4gVEVBRXMgd2VyZSBnZW5lcmFsbHkgbWFuYWdlYWJsZSB3aXRoIG5vIHVuZXhwZWN0ZWQgc2FmZXR5IGZpbmRpbmdzLiAoQ2xpbmljYWxUcmlhbHMuZ292OiBOQ1QwNDYzNTYzMSBbcHJvc3BlY3RpdmVseSByZWdpc3RlcmVkIE5vdmVtYmVyIDE5LCAyMDIwXSk8L3N0eWxlPjwvYWJzdHJhY3Q%2BPHVybHM%2BPHJlbGF0ZWQtdXJscz48dXJsPjxzdHlsZSBzaXplPVwiMTAwJVwiIGZvbnQ9XCJkZWZhdWx0XCI%2BaHR0cHM6Ly9saW5rLnNwcmluZ2VyLmNvbS8xMC4xMDA3L3MxMDYzNy0wMjMtMDEzNTEtdzwvc3R5bGU%2BPC91cmw%2BPC9yZWxhdGVkLXVybHM%2BPC91cmxzPjxlbGVjdHJvbmljLXJlc291cmNlLW51bT48c3R5bGUgc2l6ZT1cIjEwMCVcIiBmb250PVwiZGVmYXVsdFwiPjEwLjEwMDcvczEwNjM3LTAyMy0wMTM1MS13PC9zdHlsZT48L2VsZWN0cm9uaWMtcmVzb3VyY2UtbnVtPjxudW1iZXI%2BPHN0eWxlIHNpemU9XCIxMDAlXCIgZm9udD1cImRlZmF1bHRcIj4zPC9zdHlsZT48L251bWJlcj48cmVjLWd1aWQ%2BNDQxMzU1MzYtMmVlNS00YmMxLWJjNjItY2ZmZjgzZjc5OGQ4PC9yZWMtZ3VpZD48cmVjLXVzbj4zMTwvcmVjLXVzbj48L3JlY29yZD4iLCJhdXRob3JzIjpbIkx1bywgWWFuZyIsIkNoZW5nLCBZaW5nIiwiV3UsIENodW5qaWFvIiwiWWUsIEh1aSIsIkNoZW4sIE5haWhhbiIsIlpoYW5nLCBGYW4iLCJXZWksIEh1YSIsIlh1LCBCaW5naGUiXSwidGl0bGUiOiJQaGFybWFjb2tpbmV0aWNzLCBzYWZldHksIGFuZCBhbnRpdHVtb3IgYWN0aXZpdHkgb2YgdGFsYXpvcGFyaWIgbW9ub3RoZXJhcHkgaW4gQ2hpbmVzZSBwYXRpZW50cyB3aXRoIGFkdmFuY2VkIHNvbGlkIHR1bW9ycyIsImd1aWQiOiI0NDEzNTUzNi0yZWU1LTRiYzEtYmM2Mi1jZmZmODNmNzk4ZDgiLCJyZWNvcmRTdGF0dXMiOiJhY3RpdmUiLCJlbGVjdHJvbmljUmVzb3VyY2VOdW1iZXIiOiIxMC4xMDA3L3MxMDYzNy0wMjMtMDEzNTEtdyIsImdyb3VwR3VpZHMiOltdLCJ2b2x1bWUiOiI0MSIsInBhZ2VzIjoiNTAzLTUxMSJ9XSwicmVjb3JkIjp7ImNvbnRyaWJ1dG9ycyI6eyJhdXRob3JzIjp7ImF1dGhvciI6WyJMdW8sIFlhbmciLCJDaGVuZywgWWluZyIsIld1LCBDaHVuamlhbyIsIlllLCBIdWkiLCJDaGVuLCBOYWloYW4iLCJaaGFuZywgRmFuIiwiV2VpLCBIdWEiLCJYdSwgQmluZ2hlIl19fSwicmVjLXVzbiI6IjMxIiwiYWJzdHJhY3QiOiJUYWxhem9wYXJpYiwgYSBwb2x5KEFEUC1yaWJvc2UpIHBvbHltZXJhc2UgaW5oaWJpdG9yLCBoYXMgZGVtb25zdHJhdGVkIGVmZmljYWN5IGluIHRoZSB0cmVhdG1lbnQgb2YgYWR2YW5jZWQgYnJlYXN0IGFuZCBwcm9zdGF0ZSBjYW5jZXJzIGluIFdlc3Rlcm4gcG9wdWxhdGlvbnMuIFRoaXMgb3Blbi1sYWJlbCwgcGhhc2UgMSBzdHVkeSBpbnZlc3RpZ2F0ZWQgdGhlIHBoYXJtYWNva2luZXRpY3MsIHNhZmV0eSwgYW5kIGFudGl0dW1vciBhY3Rpdml0eSBvZiB0YWxhem9wYXJpYiBtb25vdGhlcmFweSBpbiBDaGluZXNlIHBhdGllbnRzIHdpdGggYWR2YW5jZWQgc29saWQgdHVtb3JzLiBNb2xlY3VsYXJseSB1bnNlbGVjdGVkIHBhdGllbnRzICjiiaUxOCB5ZWFycykgd2l0aCBhZHZhbmNlZCBzb2xpZCB0dW1vcnMgcmVzaXN0YW50IHRvIHN0YW5kYXJkIHRoZXJhcHkgcmVjZWl2ZWQgdGFsYXpvcGFyaWIgKG9yYWwsIDEgbWcgb25jZSBkYWlseSkuIFByaW1hcnkgZW5kcG9pbnQgd2FzIGNoYXJhY3Rlcml6YXRpb24gb2Ygc2luZ2xlLWRvc2UgYW5kIHN0ZWFkeS1zdGF0ZSBwaGFybWFjb2tpbmV0aWNzLiBTZWNvbmRhcnkgZW5kcG9pbnRzIGV2YWx1YXRlZCBzYWZldHksIHVuY29uZmlybWVkIG9iamVjdGl2ZSByZXNwb25zZSByYXRlIChPUlIpLCBhbmQgZHVyYXRpb24gb2YgcmVzcG9uc2UuIFRoZSBzYWZldHkgcG9wdWxhdGlvbiBjb21wcmlzZWQgMTUgQ2hpbmVzZSBwYXRpZW50cyAobWVkaWFuIFtyYW5nZV0gYWdlIDUzLjAgWzMxLjDigJM3Mi4wXSB5ZWFycykuIFNpbmdsZS1kb3NlIG1lZGlhbiB0aW1lIHRvIGZpcnN0IG9jY3VycmVuY2Ugb2YgbWF4aW11bSBvYnNlcnZlZCBjb25jZW50cmF0aW9uIHdhcyAxLjkgaDsgY29uY2VudHJhdGlvbnMgdGhlbiBkZWNsaW5lZCB3aXRoIGEgbWVhbiB0ZXJtaW5hbCBoYWxmLWxpZmUgKHQgMS8yICkgb2YgNjcgaC4gRm9sbG93aW5nIG11bHRpcGxlIGRvc2luZywgbWVkaWFuIFQgbWF4IHdhcyBhcHByb3hpbWF0ZWx5IDEuODUgaCB3aXRoIHN0ZWFkeSBzdGF0ZSBnZW5lcmFsbHkgYWNoaWV2ZWQgYnkgRGF5IDIxLiBUcmVhdG1lbnQtcmVsYXRlZCB0cmVhdG1lbnQtZW1lcmdlbnQgYWR2ZXJzZSBldmVudHMgKFRFQUVzKSBvY2N1cnJlZCBpbiA4Ni43JSAoMTMvMTUpIG9mIHBhdGllbnRzIChncmFkZSAzLCAyMC4wJTsgZ3JhZGUgNCwgMTMuMyUpLiBUd28gcGF0aWVudHMgKDEzLjMlKSBleHBlcmllbmNlZCBzZXJpb3VzIHRyZWF0bWVudC1yZWxhdGVkIFRFQUVzLiBPUlIgKGludmVzdGlnYXRvci1hc3Nlc3NlZCkgd2FzIDYuNyUgKDk1JSBDSTogMC4y4oCTMzEuOSk7IG9uZSBwYXRpZW50ICg2LjclKSBoYWQgYSBwYXJ0aWFsIHJlc3BvbnNlLiBJbiBwYXRpZW50cyB3aXRoIG1lYXN1cmFibGUgZGlzZWFzZSBhdCBiYXNlbGluZSwgdGhlIE9SUiB3YXMgOS4xJSAoMS8xMTsgOTUlIENJOiAwLjLigJM0MS4zOyBkdXJhdGlvbiBvZiByZXNwb25zZTogMTE0IGRheXMpOyBzdGFibGUgZGlzZWFzZSB3YXMgYWNoaWV2ZWQgYnkgMzYuNCUgKDQvMTEpIG9mIHBhdGllbnRzLCBhbmQgNTQuNSUgKDYvMTEpIHByb2dyZXNzZWQgYnkgZGF0YSBjdXQtb2ZmLiBJbiBDaGluZXNlIHBhdGllbnRzIHdpdGggYWR2YW5jZWQgc29saWQgdHVtb3JzLCB0aGUgcGhhcm1hY29raW5ldGljIHByb2ZpbGUgb2YgdGFsYXpvcGFyaWIgbW9ub3Ro) | - 1. μM   (0.05) | >20 μM | >20 μM | >20 μM |
| Gemcitabine | 9.1-15.6 μg/ml | [[7, 8]](https://web.endnote.com/citations/eyJkaXNwbGF5VGV4dCI6Ils3LCA4XSIsImNpdGF0aW9ucyI6W3sicmVjb3JkIjp7InZvbHVtZSI6IjY1IiwicmVmLXR5cGUiOiIxNyIsInRpdGxlcyI6eyJzZWNvbmRhcnktdGl0bGUiOiJDYW5jZXIgQ2hlbW90aGVyYXB5IGFuZCBQaGFybWFjb2xvZ3kiLCJ0aXRsZSI6IlBoYXJtYWNva2luZXRpYyBzdHVkeSBvZiBnZW1jaXRhYmluZSwgZ2l2ZW4gYXMgcHJvbG9uZ2VkIGluZnVzaW9uIGF0IGZpeGVkIGRvc2UgcmF0ZSwgaW4gY29tYmluYXRpb24gd2l0aCBjaXNwbGF0aW4gaW4gcGF0aWVudHMgd2l0aCBhZHZhbmNlZCBub24tc21hbGwtY2VsbCBsdW5nIGNhbmNlciJ9LCJ1cmxzIjp7InJlbGF0ZWQtdXJscyI6eyJ1cmwiOiJodHRwOi8vbGluay5zcHJpbmdlci5jb20vMTAuMTAwNy9zMDAyODAtMDEwLTEyNTUtNyJ9fSwiZWxlY3Ryb25pYy1yZXNvdXJjZS1udW0iOiIxMC4xMDA3L3MwMDI4MC0wMTAtMTI1NS03IiwiZGF0ZXMiOnsieWVhciI6IjIwMTAifSwicmVjLXVzbiI6IjQ1IiwibnVtYmVyIjoiNiIsImNvbnRyaWJ1dG9ycyI6eyJhdXRob3JzIjp7ImF1dGhvciI6WyJDYWZmbywgT3JhemlvIiwiRmFsbGFuaSwgU3RlZmFuaWEiLCJNYXJhbmdvbiwgRWxlbmEiLCJOb2JpbGksIFN0ZWZhbmlhIiwiQ2Fzc2V0dGEsIE1hcmlhIElyaXMiLCJNdXJnaWEsIFZpdmlhbmEiLCJTYWxhLCBGZWRlcmljYSIsIk5vdmVsbGksIEFuZHJlYSIsIk1pbmksIEVucmljbyIsIlp1Y2NoZXR0aSwgTWFzc2ltbyIsIkdhbGxpZ2lvbmksIEVuem8iXX19LCJwYWdlcyI6IjExOTctMTIwMiIsInJlYy1ndWlkIjoiMTE2OGNhMzktMGI3OS00YTU5LWFmNWYtODFiYWQzZjU4Y2MyIn0sImJpYmxpb0NvbnRlbnQiOlt7Imd1aWQiOiIxMTY4Y2EzOS0wYjc5LTRhNTktYWY1Zi04MWJhZDNmNThjYzIiLCJ1cmwiOlsiaHR0cDovL2xpbmsuc3ByaW5nZXIuY29tLzEwLjEwMDcvczAwMjgwLTAxMC0xMjU1LTciXSwiZ3JvdXBHdWlkcyI6W10sInRpdGxlIjoiUGhhcm1hY29raW5ldGljIHN0dWR5IG9mIGdlbWNpdGFiaW5lLCBnaXZlbiBhcyBwcm9sb25nZWQgaW5mdXNpb24gYXQgZml4ZWQgZG9zZSByYXRlLCBpbiBjb21iaW5hdGlvbiB3aXRoIGNpc3BsYXRpbiBpbiBwYXRpZW50cyB3aXRoIGFkdmFuY2VkIG5vbi1zbWFsbC1jZWxsIGx1bmcgY2FuY2VyIiwidm9sdW1lIjoiNjUiLCJudW1iZXIiOiI2IiwiZWxlY3Ryb25pY1Jlc291cmNlTnVtYmVyIjoiMTAuMTAwNy9zMDAyODAtMDEwLTEyNTUtNyIsInJzeG1sIjoiPHJlY29yZD48cmVmLXR5cGU%2BMTc8L3JlZi10eXBlPjxjb250cmlidXRvcnM%2BPGF1dGhvcnM%2BPGF1dGhvcj48c3R5bGUgc2l6ZT1cIjEwMCVcIiBmb250PVwiZGVmYXVsdFwiPkNhZmZvLCBPcmF6aW88L3N0eWxlPjwvYXV0aG9yPjxhdXRob3I%2BPHN0eWxlIHNpemU9XCIxMDAlXCIgZm9udD1cImRlZmF1bHRcIj5GYWxsYW5pLCBTdGVmYW5pYTwvc3R5bGU%2BPC9hdXRob3I%2BPGF1dGhvcj48c3R5bGUgc2l6ZT1cIjEwMCVcIiBmb250PVwiZGVmYXVsdFwiPk1hcmFuZ29uLCBFbGVuYTwvc3R5bGU%2BPC9hdXRob3I%2BPGF1dGhvcj48c3R5bGUgc2l6ZT1cIjEwMCVcIiBmb250PVwiZGVmYXVsdFwiPk5vYmlsaSwgU3RlZmFuaWE8L3N0eWxlPjwvYXV0aG9yPjxhdXRob3I%2BPHN0eWxlIHNpemU9XCIxMDAlXCIgZm9udD1cImRlZmF1bHRcIj5DYXNzZXR0YSwgTWFyaWEgSXJpczwvc3R5bGU%2BPC9hdXRob3I%2BPGF1dGhvcj48c3R5bGUgc2l6ZT1cIjEwMCVcIiBmb250PVwiZGVmYXVsdFwiPk11cmdpYSwgVml2aWFuYTwvc3R5bGU%2BPC9hdXRob3I%2BPGF1dGhvcj48c3R5bGUgc2l6ZT1cIjEwMCVcIiBmb250PVwiZGVmYXVsdFwiPlNhbGEsIEZlZGVyaWNhPC9zdHlsZT48L2F1dGhvcj48YXV0aG9yPjxzdHlsZSBzaXplPVwiMTAwJVwiIGZvbnQ9XCJkZWZhdWx0XCI%2BTm92ZWxsaSwgQW5kcmVhPC9zdHlsZT48L2F1dGhvcj48YXV0aG9yPjxzdHlsZSBzaXplPVwiMTAwJVwiIGZvbnQ9XCJkZWZhdWx0XCI%2BTWluaSwgRW5yaWNvPC9zdHlsZT48L2F1dGhvcj48YXV0aG9yPjxzdHlsZSBzaXplPVwiMTAwJVwiIGZvbnQ9XCJkZWZhdWx0XCI%2BWnVjY2hldHRpLCBNYXNzaW1vPC9zdHlsZT48L2F1dGhvcj48YXV0aG9yPjxzdHlsZSBzaXplPVwiMTAwJVwiIGZvbnQ9XCJkZWZhdWx0XCI%2BR2FsbGlnaW9uaSwgRW56bzwvc3R5bGU%2BPC9hdXRob3I%2BPC9hdXRob3JzPjwvY29udHJpYnV0b3JzPjx0aXRsZXM%2BPHRpdGxlPjxzdHlsZSBzaXplPVwiMTAwJVwiIGZvbnQ9XCJkZWZhdWx0XCI%2BUGhhcm1hY29raW5ldGljIHN0dWR5IG9mIGdlbWNpdGFiaW5lLCBnaXZlbiBhcyBwcm9sb25nZWQgaW5mdXNpb24gYXQgZml4ZWQgZG9zZSByYXRlLCBpbiBjb21iaW5hdGlvbiB3aXRoIGNpc3BsYXRpbiBpbiBwYXRpZW50cyB3aXRoIGFkdmFuY2VkIG5vbi1zbWFsbC1jZWxsIGx1bmcgY2FuY2VyPC9zdHlsZT48L3RpdGxlPjxzZWNvbmRhcnktdGl0bGU%2BPHN0eWxlIHNpemU9XCIxMDAlXCIgZm9udD1cImRlZmF1bHRcIj5DYW5jZXIgQ2hlbW90aGVyYXB5IGFuZCBQaGFybWFjb2xvZ3k8L3N0eWxlPjwvc2Vjb25kYXJ5LXRpdGxlPjwvdGl0bGVzPjxkYXRlcz48eWVhcj48c3R5bGUgc2l6ZT1cIjEwMCVcIiBmb250PVwiZGVmYXVsdFwiPjIwMTA8L3N0eWxlPjwveWVhcj48L2RhdGVzPjxwYWdlcz48c3R5bGUgc2l6ZT1cIjEwMCVcIiBmb250PVwiZGVmYXVsdFwiPjExOTctMTIwMjwvc3R5bGU%2BPC9wYWdlcz48dm9sdW1lPjxzdHlsZSBzaXplPVwiMTAwJVwiIGZvbnQ9XCJkZWZhdWx0XCI%2BNjU8L3N0eWxlPjwvdm9sdW1lPjx1cmxzPjxyZWxhdGVkLXVybHM%2BPHVybD48c3R5bGUgc2l6ZT1cIjEwMCVcIiBmb250PVwiZGVmYXVsdFwiPmh0dHA6Ly9saW5rLnNwcmluZ2VyLmNvbS8xMC4xMDA3L3MwMDI4MC0wMTAtMTI1NS03PC9zdHlsZT48L3VybD48L3JlbGF0ZWQtdXJscz48L3VybHM%2BPGVsZWN0cm9uaWMtcmVzb3VyY2UtbnVtPjxzdHlsZSBzaXplPVwiMTAwJVwiIGZvbnQ9XCJkZWZhdWx0XCI%2BMTAuMTAwNy9zMDAyODAtMDEwLTEyNTUtNzwvc3R5bGU%2BPC9lbGVjdHJvbmljLXJlc291cmNlLW51bT48bnVtYmVyPjxzdHlsZSBzaXplPVwiMTAwJVwiIGZvbnQ9XCJkZWZhdWx0XCI%2BNjwvc3R5bGU%2BPC9udW1iZXI%2BPHJlYy1ndWlkPjExNjhjYTM5LTBiNzktNGE1OS1hZjVmLTgxYmFkM2Y1OGNjMjwvcmVjLWd1aWQ%2BPHJlYy11c24%2BNDU8L3JlYy11c24%2BPC9yZWNvcmQ%2BIiwicGFnZXMiOiIxMTk3LTEyMDIiLCJ5ZWFyIjoiMjAxMCIsInJlZmVyZW5jZVR5cGUiOiIxNyIsInNlY29uZGFyeVRpdGxlIjoiQ2FuY2VyIENoZW1vdGhlcmFweSBhbmQgUGhhcm1hY29sb2d5IiwicmVjb3JkU3RhdHVzIjoiYWN0aXZlIiwiYXV0aG9ycyI6WyJDYWZmbywgT3JhemlvIiwiRmFsbGFuaSwgU3RlZmFuaWEiLCJNYXJhbmdvbiwgRWxlbmEiLCJOb2JpbGksIFN0ZWZhbmlhIiwiQ2Fzc2V0dGEsIE1hcmlhIElyaXMiLCJNdXJnaWEsIFZpdmlhbmEiLCJTYWxhLCBGZWRlcmljYSIsIk5vdmVsbGksIEFuZHJlYSIsIk1pbmksIEVucmljbyIsIlp1Y2NoZXR0aSwgTWFzc2ltbyIsIkdhbGxpZ2lvbmksIEVuem8iXX1dLCJndWlkIjoiMTE2OGNhMzktMGI3OS00YTU5LWFmNWYtODFiYWQzZjU4Y2MyIn0seyJndWlkIjoiOGY0MDFkMDItNjY1My00OWFiLTk4NWQtNzk3NzZjYTY0YWIzIiwiYmlibGlvQ29udGVudCI6W3sieWVhciI6IjIwMDgiLCJzZWNvbmRhcnlUaXRsZSI6IkphcGFuZXNlIEpvdXJuYWwgb2YgQ2xpbmljYWwgT25jb2xvZ3kiLCJyc3htbCI6IjxyZWNvcmQ%2BPHJlZi10eXBlPjE3PC9yZWYtdHlwZT48Y29udHJpYnV0b3JzPjxhdXRob3JzPjxhdXRob3I%2BPHN0eWxlIHNpemU9XCIxMDAlXCIgZm9udD1cImRlZmF1bHRcIj5NYXN1bW9yaSwgTi48L3N0eWxlPjwvYXV0aG9yPjxhdXRob3I%2BPHN0eWxlIHNpemU9XCIxMDAlXCIgZm9udD1cImRlZmF1bHRcIj5LdW5pc2hpbWEsIFkuPC9zdHlsZT48L2F1dGhvcj48YXV0aG9yPjxzdHlsZSBzaXplPVwiMTAwJVwiIGZvbnQ9XCJkZWZhdWx0XCI%2BSGlyb2JlLCBNLjwvc3R5bGU%2BPC9hdXRob3I%2BPGF1dGhvcj48c3R5bGUgc2l6ZT1cIjEwMCVcIiBmb250PVwiZGVmYXVsdFwiPlRha2V1Y2hpLCBNLjwvc3R5bGU%2BPC9hdXRob3I%2BPGF1dGhvcj48c3R5bGUgc2l6ZT1cIjEwMCVcIiBmb250PVwiZGVmYXVsdFwiPlRha2F5YW5hZ2ksIEEuPC9zdHlsZT48L2F1dGhvcj48YXV0aG9yPjxzdHlsZSBzaXplPVwiMTAwJVwiIGZvbnQ9XCJkZWZhdWx0XCI%2BVHN1a2Ftb3RvLCBULjwvc3R5bGU%2BPC9hdXRob3I%2BPGF1dGhvcj48c3R5bGUgc2l6ZT1cIjEwMCVcIiBmb250PVwiZGVmYXVsdFwiPkl0b2gsIFQuPC9zdHlsZT48L2F1dGhvcj48L2F1dGhvcnM%2BPC9jb250cmlidXRvcnM%2BPHRpdGxlcz48dGl0bGU%2BPHN0eWxlIHNpemU9XCIxMDAlXCIgZm9udD1cImRlZmF1bHRcIj5NZWFzdXJlbWVudCBvZiBQbGFzbWEgQ29uY2VudHJhdGlvbiBvZiBHZW1jaXRhYmluZSBhbmQgSXRzIE1ldGFib2xpdGUgZEZkVSBpbiBIZW1vZGlhbHlzaXMgUGF0aWVudHMgd2l0aCBBZHZhbmNlZCBVcm90aGVsaWFsIENhbmNlcjwvc3R5bGU%2BPC90aXRsZT48c2Vjb25kYXJ5LXRpdGxlPjxzdHlsZSBzaXplPVwiMTAwJVwiIGZvbnQ9XCJkZWZhdWx0XCI%2BSmFwYW5lc2UgSm91cm5hbCBvZiBDbGluaWNhbCBPbmNvbG9neTwvc3R5bGU%2BPC9zZWNvbmRhcnktdGl0bGU%2BPC90aXRsZXM%2BPGRhdGVzPjx5ZWFyPjxzdHlsZSBzaXplPVwiMTAwJVwiIGZvbnQ9XCJkZWZhdWx0XCI%2BMjAwODwvc3R5bGU%2BPC95ZWFyPjwvZGF0ZXM%2BPHBhZ2VzPjxzdHlsZSBzaXplPVwiMTAwJVwiIGZvbnQ9XCJkZWZhdWx0XCI%2BMTgyLTE4NTwvc3R5bGU%2BPC9wYWdlcz48dm9sdW1lPjxzdHlsZSBzaXplPVwiMTAwJVwiIGZvbnQ9XCJkZWZhdWx0XCI%2BMzg8L3N0eWxlPjwvdm9sdW1lPjx1cmxzPjxyZWxhdGVkLXVybHM%2BPHVybD48c3R5bGUgc2l6ZT1cIjEwMCVcIiBmb250PVwiZGVmYXVsdFwiPmh0dHBzOi8vYWNhZGVtaWMub3VwLmNvbS9qamNvL2FydGljbGUtbG9va3VwL2RvaS8xMC4xMDkzL2pqY28vaHltMTcxPC9zdHlsZT48L3VybD48L3JlbGF0ZWQtdXJscz48L3VybHM%2BPGVsZWN0cm9uaWMtcmVzb3VyY2UtbnVtPjxzdHlsZSBzaXplPVwiMTAwJVwiIGZvbnQ9XCJkZWZhdWx0XCI%2BMTAuMTA5My9qamNvL2h5bTE3MTwvc3R5bGU%2BPC9lbGVjdHJvbmljLXJlc291cmNlLW51bT48bnVtYmVyPjxzdHlsZSBzaXplPVwiMTAwJVwiIGZvbnQ9XCJkZWZhdWx0XCI%2BMzwvc3R5bGU%2BPC9udW1iZXI%2BPHJlYy1ndWlkPjhmNDAxZDAyLTY2NTMtNDlhYi05ODVkLTc5Nzc2Y2E2NGFiMzwvcmVjLWd1aWQ%2BPHJlYy11c24%2BNDA8L3JlYy11c24%2BPC9yZWNvcmQ%2BIiwidXJsIjpbImh0dHBzOi8vYWNhZGVtaWMub3VwLmNvbS9qamNvL2FydGljbGUtbG9va3VwL2RvaS8xMC4xMDkzL2pqY28vaHltMTcxIl0sInBhZ2VzIjoiMTgyLTE4NSIsIm51bWJlciI6IjMiLCJ0aXRsZSI6Ik1lYXN1cmVtZW50IG9mIFBsYXNtYSBDb25jZW50cmF0aW9uIG9mIEdlbWNpdGFiaW5lIGFuZCBJdHMgTWV0YWJvbGl0ZSBkRmRVIGluIEhlbW9kaWFseXNpcyBQYXRpZW50cyB3aXRoIEFkdmFuY2VkIFVyb3RoZWxpYWwgQ2FuY2VyIiwidm9sdW1lIjoiMzgiLCJndWlkIjoiOGY0MDFkMDItNjY1My00OWFiLTk4NWQtNzk3NzZjYTY0YWIzIiwiZWxlY3Ryb25pY1Jlc291cmNlTnVtYmVyIjoiMTAuMTA5My9qamNvL2h5bTE3MSIsImF1dGhvcnMiOlsiTWFzdW1vcmksIE4uIiwiS3VuaXNoaW1hLCBZLiIsIkhpcm9iZSwgTS4iLCJUYWtldWNoaSwgTS4iLCJUYWtheWFuYWdpLCBBLiIsIlRzdWthbW90bywgVC4iLCJJdG9oLCBULiJdLCJncm91cEd1aWRzIjpbXSwicmVjb3JkU3RhdHVzIjoiYWN0aXZlIiwicmVmZXJlbmNlVHlwZSI6IjE3In1dLCJyZWNvcmQiOnsicmVmLXR5cGUiOiIxNyIsImNvbnRyaWJ1dG9ycyI6eyJhdXRob3JzIjp7ImF1dGhvciI6WyJNYXN1bW9yaSwgTi4iLCJLdW5pc2hpbWEsIFkuIiwiSGlyb2JlLCBNLiIsIlRha2V1Y2hpLCBNLiIsIlRha2F5YW5hZ2ksIEEuIiwiVHN1a2Ftb3RvLCBULiIsIkl0b2gsIFQuIl19) | 0.06 ng/ml  (0.03) | 0.24 ng/ml  (0.29, 0.4) | 0.05  ng/ml  (0.075, 0.8) | 0.12 ng/ml  (0.074, 0.2) |
| 5-FU | 168-423 μM | [[9, 10]](https://web.endnote.com/citations/eyJkaXNwbGF5VGV4dCI6Ils5LCAxMF0iLCJjaXRhdGlvbnMiOlt7InJlY29yZCI6eyJ0aXRsZXMiOnsic2Vjb25kYXJ5LXRpdGxlIjoiUGhhcm1hY29sb2dpY2FsIHJlc2VhcmNoIiwidGl0bGUiOiJQbGFzbWEgY29uY2VudHJhdGlvbnMgb2YgNS1mbHVvcm91cmFjaWwgYW5kIGl0cyBtZXRhYm9saXRlcyBpbiBjb2xvbiBjYW5jZXIgcGF0aWVudHMifSwicmVjLWd1aWQiOiJhMjljMDJmZi03NTM0LTQ4YzYtYTY5Zi1mMjg5OTU5NDQ2OGMiLCJudW1iZXIiOiIyIiwidXJscyI6eyJyZWxhdGVkLXVybHMiOnsidXJsIjoiaHR0cHM6Ly9wdWJtZWQubmNiaS5ubG0ubmloLmdvdi8xNTE3NzMwNi8ifX0sImRhdGVzIjp7InllYXIiOiIyMDA0In0sImNvbnRyaWJ1dG9ycyI6eyJhdXRob3JzIjp7ImF1dGhvciI6WyJGZWRlcmljbyBDYXNhbGUgIiwiUm9iZXJ0byBDYW5hcGFybyIsIkxvcmVkYW5hIFNlcnBlIiwiRWxpc2FiZXR0YSBNdW50b25pIiwiQ2FybG8gRGVsbGEgUGVwYSIsIk1hcmlvIENvc3RhIiwiTG9yZW56YSBNYWlyb25lIiwiR2lhbiBQYW9sbyBaYXJhIiwiR2lhbm5pIEZvcm5hcmkiLCJNYXJpbyBFYW5kaSJdfX0sImFic3RyYWN0IjoiNS1GbHVvcm91cmFjaWwgKDUtRlUpIGlzIGEgY29tbW9uIGFudGljYW5jZXIgYWdlbnQgdXNlZCBpbiB0aGUgdHJlYXRtZW50IG9mIHNvbGlkIHR1bW91cnMsIHdpdGggYSByZXBvcnRlZCB2YXJpYWJpbGl0eSBpbiB0aGUgcGhhcm1hY29raW5ldGljIHByb2ZpbGUgYW5kIGludGVyLXBhdGllbnQgZGlmZmVyZW5jZXMgaW4gZWZmaWNhY3kgYW5kIHRveGljaXR5LiBTaW5jZSA1LUZVIGlzIGludHJhY2VsbHVsYXJseSBtZXRhYm9saXNlZCB0byBhY3RpdmUgY3l0b3RveGljIGZsdW9yb251Y2xlb3RpZGVzLCBzb21lIGF1dGhvcnMg4oCmIiwicmVjLXVzbiI6IjEwNTgiLCJpc2JuIjoiMTA0My02NjE4Iiwidm9sdW1lIjoiNTAiLCJyZWYtdHlwZSI6IjE3IiwiZWxlY3Ryb25pYy1yZXNvdXJjZS1udW0iOiIxMC4xMDE2L2oucGhycy4yMDA0LjAxLjAwNiJ9LCJndWlkIjoiYTI5YzAyZmYtNzUzNC00OGM2LWE2OWYtZjI4OTk1OTQ0NjhjIiwiYmlibGlvQ29udGVudCI6W3siZ3VpZCI6ImEyOWMwMmZmLTc1MzQtNDhjNi1hNjlmLWYyODk5NTk0NDY4YyIsInVybCI6WyJodHRwczovL3B1Ym1lZC5uY2JpLm5sbS5uaWguZ292LzE1MTc3MzA2LyJdLCJncm91cEd1aWRzIjpbXSwibnVtYmVyIjoiMiIsInJzeG1sIjoiPHJlY29yZD48cmVmLXR5cGU%2BMTc8L3JlZi10eXBlPjxjb250cmlidXRvcnM%2BPGF1dGhvcnM%2BPGF1dGhvcj48c3R5bGUgc2l6ZT1cIjEwMCVcIiBmb250PVwiZGVmYXVsdFwiPkZlZGVyaWNvIENhc2FsZSA8L3N0eWxlPjwvYXV0aG9yPjxhdXRob3I%2BPHN0eWxlIHNpemU9XCIxMDAlXCIgZm9udD1cImRlZmF1bHRcIj5Sb2JlcnRvIENhbmFwYXJvPC9zdHlsZT48L2F1dGhvcj48YXV0aG9yPjxzdHlsZSBzaXplPVwiMTAwJVwiIGZvbnQ9XCJkZWZhdWx0XCI%2BTG9yZWRhbmEgU2VycGU8L3N0eWxlPjwvYXV0aG9yPjxhdXRob3I%2BPHN0eWxlIHNpemU9XCIxMDAlXCIgZm9udD1cImRlZmF1bHRcIj5FbGlzYWJldHRhIE11bnRvbmk8L3N0eWxlPjwvYXV0aG9yPjxhdXRob3I%2BPHN0eWxlIHNpemU9XCIxMDAlXCIgZm9udD1cImRlZmF1bHRcIj5DYXJsbyBEZWxsYSBQZXBhPC9zdHlsZT48L2F1dGhvcj48YXV0aG9yPjxzdHlsZSBzaXplPVwiMTAwJVwiIGZvbnQ9XCJkZWZhdWx0XCI%2BTWFyaW8gQ29zdGE8L3N0eWxlPjwvYXV0aG9yPjxhdXRob3I%2BPHN0eWxlIHNpemU9XCIxMDAlXCIgZm9udD1cImRlZmF1bHRcIj5Mb3JlbnphIE1haXJvbmU8L3N0eWxlPjwvYXV0aG9yPjxhdXRob3I%2BPHN0eWxlIHNpemU9XCIxMDAlXCIgZm9udD1cImRlZmF1bHRcIj5HaWFuIFBhb2xvIFphcmE8L3N0eWxlPjwvYXV0aG9yPjxhdXRob3I%2BPHN0eWxlIHNpemU9XCIxMDAlXCIgZm9udD1cImRlZmF1bHRcIj5HaWFubmkgRm9ybmFyaTwvc3R5bGU%2BPC9hdXRob3I%2BPGF1dGhvcj48c3R5bGUgc2l6ZT1cIjEwMCVcIiBmb250PVwiZGVmYXVsdFwiPk1hcmlvIEVhbmRpPC9zdHlsZT48L2F1dGhvcj48L2F1dGhvcnM%2BPC9jb250cmlidXRvcnM%2BPHRpdGxlcz48dGl0bGU%2BPHN0eWxlIHNpemU9XCIxMDAlXCIgZm9udD1cImRlZmF1bHRcIj5QbGFzbWEgY29uY2VudHJhdGlvbnMgb2YgNS1mbHVvcm91cmFjaWwgYW5kIGl0cyBtZXRhYm9saXRlcyBpbiBjb2xvbiBjYW5jZXIgcGF0aWVudHM8L3N0eWxlPjwvdGl0bGU%2BPHNlY29uZGFyeS10aXRsZT48c3R5bGUgc2l6ZT1cIjEwMCVcIiBmb250PVwiZGVmYXVsdFwiPlBoYXJtYWNvbG9naWNhbCByZXNlYXJjaDwvc3R5bGU%2BPC9zZWNvbmRhcnktdGl0bGU%2BPC90aXRsZXM%2BPGRhdGVzPjx5ZWFyPjxzdHlsZSBzaXplPVwiMTAwJVwiIGZvbnQ9XCJkZWZhdWx0XCI%2BMjAwNDwvc3R5bGU%2BPC95ZWFyPjwvZGF0ZXM%2BPHZvbHVtZT48c3R5bGUgc2l6ZT1cIjEwMCVcIiBmb250PVwiZGVmYXVsdFwiPjUwPC9zdHlsZT48L3ZvbHVtZT48aXNibj48c3R5bGUgc2l6ZT1cIjEwMCVcIiBmb250PVwiZGVmYXVsdFwiPjEwNDMtNjYxODwvc3R5bGU%2BPC9pc2JuPjxhYnN0cmFjdD48c3R5bGUgc2l6ZT1cIjEwMCVcIiBmb250PVwiZGVmYXVsdFwiPjUtRmx1b3JvdXJhY2lsICg1LUZVKSBpcyBhIGNvbW1vbiBhbnRpY2FuY2VyIGFnZW50IHVzZWQgaW4gdGhlIHRyZWF0bWVudCBvZiBzb2xpZCB0dW1vdXJzLCB3aXRoIGEgcmVwb3J0ZWQgdmFyaWFiaWxpdHkgaW4gdGhlIHBoYXJtYWNva2luZXRpYyBwcm9maWxlIGFuZCBpbnRlci1wYXRpZW50IGRpZmZlcmVuY2VzIGluIGVmZmljYWN5IGFuZCB0b3hpY2l0eS4gU2luY2UgNS1GVSBpcyBpbnRyYWNlbGx1bGFybHkgbWV0YWJvbGlzZWQgdG8gYWN0aXZlIGN5dG90b3hpYyBmbHVvcm9udWNsZW90aWRlcywgc29tZSBhdXRob3JzIOKApjwvc3R5bGU%2BPC9hYnN0cmFjdD48dXJscz48cmVsYXRlZC11cmxzPjx1cmw%2BPHN0eWxlIHNpemU9XCIxMDAlXCIgZm9udD1cImRlZmF1bHRcIj5odHRwczovL3B1Ym1lZC5uY2JpLm5sbS5uaWguZ292LzE1MTc3MzA2Lzwvc3R5bGU%2BPC91cmw%2BPC9yZWxhdGVkLXVybHM%2BPC91cmxzPjxlbGVjdHJvbmljLXJlc291cmNlLW51bT48c3R5bGUgc2l6ZT1cIjEwMCVcIiBmb250PVwiZGVmYXVsdFwiPjEwLjEwMTYvai5waHJzLjIwMDQuMDEuMDA2PC9zdHlsZT48L2VsZWN0cm9uaWMtcmVzb3VyY2UtbnVtPjxudW1iZXI%2BPHN0eWxlIHNpemU9XCIxMDAlXCIgZm9udD1cImRlZmF1bHRcIj4yPC9zdHlsZT48L251bWJlcj48cmVjLWd1aWQ%2BYTI5YzAyZmYtNzUzNC00OGM2LWE2OWYtZjI4OTk1OTQ0NjhjPC9yZWMtZ3VpZD48cmVjLXVzbj4xMDU4PC9yZWMtdXNuPjwvcmVjb3JkPiIsInZvbHVtZSI6IjUwIiwiYXV0aG9ycyI6WyJGZWRlcmljbyBDYXNhbGUgIiwiUm9iZXJ0byBDYW5hcGFybyIsIkxvcmVkYW5hIFNlcnBlIiwiRWxpc2FiZXR0YSBNdW50b25pIiwiQ2FybG8gRGVsbGEgUGVwYSIsIk1hcmlvIENvc3RhIiwiTG9yZW56YSBNYWlyb25lIiwiR2lhbiBQYW9sbyBaYXJhIiwiR2lhbm5pIEZvcm5hcmkiLCJNYXJpbyBFYW5kaSJdLCJlbGVjdHJvbmljUmVzb3VyY2VOdW1iZXIiOiIxMC4xMDE2L2oucGhycy4yMDA0LjAxLjAwNiIsInJlZmVyZW5jZVR5cGUiOiIxNyIsInllYXIiOiIyMDA0IiwiaXNibiI6IjEwNDMtNjYxOCIsInRpdGxlIjoiUGxhc21hIGNvbmNlbnRyYXRpb25zIG9mIDUtZmx1b3JvdXJhY2lsIGFuZCBpdHMgbWV0YWJvbGl0ZXMgaW4gY29sb24gY2FuY2VyIHBhdGllbnRzIiwic2Vjb25kYXJ5VGl0bGUiOiJQaGFybWFjb2xvZ2ljYWwgcmVzZWFyY2giLCJyZWNvcmRTdGF0dXMiOiJhY3RpdmUifV19LHsicmVjb3JkIjp7InRpdGxlcyI6eyJ0aXRsZSI6IjUtRmx1b3JvdXJhY2lsIFBoYXJtYWNva2luZXRpY3MgUHJlZGljdHMgRGlzZWFzZS1mcmVlIFN1cnZpdmFsIGluIFBhdGllbnRzIEFkbWluaXN0ZXJlZCBBZGp1dmFudCBDaGVtb3RoZXJhcHkgZm9yIENvbG9yZWN0YWwgQ2FuY2VyIiwic2Vjb25kYXJ5LXRpdGxlIjoiQ2xpbmljYWwgQ2FuY2VyIFJlc2VhcmNoIn0sInJlZi10eXBlIjoiMTciLCJ1cmxzIjp7InJlbGF0ZWQtdXJscyI6eyJ1cmwiOiJodHRwczovL2FhY3Jqb3VybmFscy5vcmcvY2xpbmNhbmNlcnJlcy9hcnRpY2xlLzE0LzkvMjc0OS83MzU1Ny81LUZsdW9yb3VyYWNpbC1QaGFybWFjb2tpbmV0aWNzLVByZWRpY3RzLURpc2Vhc2UifX0sInBhZ2VzIjoiMjc0OS0yNzU1Iiwidm9sdW1lIjoiMTQiLCJkYXRlcyI6eyJ5ZWFyIjoiMjAwOCJ9LCJyZWMtZ3VpZCI6ImZkY2E5MDVjLWFhNzMtNDgyMS1iN2FhLTEzNjAzMWRkZTk0YiIsIm51bWJlciI6IjkiLCJyZWMtdXNuIjoiMzMiLCJhYnN0cmFjdCI6IlB1cnBvc2U6IFRvIGV2YWx1YXRlIDUtZmx1b3JvdXJhY2lsICg1LUZVKSBhbmQgNS1mbHVvcm8tNSw2LWRpaHlkcm91cmFjaWwgKDUtRkRIVSkgcGhhcm1hY29raW5ldGljcyBhbmQgZGlzZWFzZS1mcmVlIHN1cnZpdmFsIChERlMpIGluIGNvbG9yZWN0YWwgY2FuY2VyIHBhdGllbnRzIGdpdmVuIDUtRlXigJNiYXNlZCBhZGp1dmFudCBjaGVtb3RoZXJhcHkgd2l0aGluIGEgbm9ucmFuZG9taXplZCwgcmV0cm9zcGVjdGl2ZSwgcGhhcm1hY29raW5ldGljIHN0dWR5LiIsImVsZWN0cm9uaWMtcmVzb3VyY2UtbnVtIjoiMTAuMTE1OC8xMDc4LTA0MzIuQ0NSLTA3LTE1MjkiLCJjb250cmlidXRvcnMiOnsiYXV0aG9ycyI6eyJhdXRob3IiOlsiRGkgUGFvbG8sIEFudG9uZWxsbyIsIkxlbmNpb25pLCBNb25pY2EiLCJBbWF0b3JpLCBGZWRlcmljYSIsIkRpIERvbmF0bywgU2FtYW50aGEiLCJCb2NjaSwgR3VpZG8iLCJPcmxhbmRpbmksIENpbnppYSIsIkxhc3RlbGxhLCBNYXJpYW5uYSIsIkZlZGVyaWNpLCBGcmFuY2VzY2EiLCJJYW5ub3BvbGxvLCBNYXVybyIsIkZhbGNvbmUsIEFsZnJlZG8iLCJSaWNjaSwgU2VyZ2lvIiwiRGVsIFRhY2NhLCBNYXJpbyIsIkRhbmVzaSwgUm9tYW5vIl19fX0sImd1aWQiOiJmZGNhOTA1Yy1hYTczLTQ4MjEtYjdhYS0xMzYwMzFkZGU5NGIiLCJiaWJsaW9Db250ZW50IjpbeyJ0aXRsZSI6IjUtRmx1b3JvdXJhY2lsIFBoYXJtYWNva2luZXRpY3MgUHJlZGljdHMgRGlzZWFzZS1mcmVlIFN1cnZpdmFsIGluIFBhdGllbnRzIEFkbWluaXN0ZXJlZCBBZGp1dmFudCBDaGVtb3RoZXJhcHkgZm9yIENvbG9yZWN0YWwgQ2FuY2VyIiwic2Vjb25kYXJ5VGl0bGUiOiJDbGluaWNhbCBDYW5jZXIgUmVzZWFyY2giLCJyZWZlcmVuY2VUeXBlIjoiMTciLCJlbGVjdHJvbmljUmVzb3VyY2VOdW1iZXIiOiIxMC4xMTU4LzEwNzgtMDQzMi5DQ1ItMDctMTUyOSIsInJlY29yZFN0YXR1cyI6ImFjdGl2ZSIsInZvbHVtZSI6IjE0IiwicGFnZXMiOiIyNzQ5LTI3NTUiLCJncm91cEd1aWRzIjpbXSwiZ3VpZCI6ImZkY2E5MDVjLWFhNzMtNDgyMS1iN2FhLTEzNjAzMWRkZTk0YiIsInJzeG1sIjoiPHJlY29yZD48cmVmLXR5cGU%2BMTc8L3JlZi10eXBlPjxjb250cmlidXRvcnM%2BPGF1dGhvcnM%2BPGF1dGhvcj48c3R5bGUgc2l6ZT1cIjEwMCVcIiBmb250PVwiZGVmYXVsdFwiPkRpIFBhb2xvLCBBbnRvbmVsbG88L3N0eWxlPjwvYXV0aG9yPjxhdXRob3I%2BPHN0eWxlIHNpemU9XCIxMDAlXCIgZm9udD1cImRlZmF1bHRcIj5MZW5jaW9uaSwgTW9uaWNhPC9zdHlsZT48L2F1dGhvcj48YXV0aG9yPjxzdHlsZSBzaXplPVwiMTAwJVwiIGZvbnQ9XCJkZWZhdWx0XCI%2BQW1hdG9yaSwgRmVkZXJpY2E8L3N0eWxlPjwvYXV0aG9yPjxhdXRob3I%2BPHN0eWxlIHNpemU9XCIxMDAlXCIgZm9udD1cImRlZmF1bHRcIj5EaSBEb25hdG8sIFNhbWFudGhhPC9zdHlsZT48L2F1dGhvcj48YXV0aG9yPjxzdHlsZSBzaXplPVwiMTAwJVwiIGZvbnQ9XCJkZWZhdWx0XCI%2BQm9jY2ksIEd1aWRvPC9zdHlsZT48L2F1dGhvcj48YXV0aG9yPjxzdHlsZSBzaXplPVwiMTAwJVwiIGZvbnQ9XCJkZWZhdWx0XCI%2BT3JsYW5kaW5pLCBDaW56aWE8L3N0eWxlPjwvYXV0aG9yPjxhdXRob3I%2BPHN0eWxlIHNpemU9XCIxMDAlXCIgZm9udD1cImRlZmF1bHRcIj5MYXN0ZWxsYSwgTWFyaWFubmE8L3N0eWxlPjwvYXV0aG9yPjxhdXRob3I%2BPHN0eWxlIHNpemU9XCIxMDAlXCIgZm9u) | 0.09 μM  (0.05) | 0.23 μM  (0.13, 0.1) | 0.17 μM  (1.4, 0.3) | 0.09 μM  (0.089, 0.8) |
| Oxaliplatin | 1.8-9 µM | [[11-13]](https://web.endnote.com/citations/eyJkaXNwbGF5VGV4dCI6IlsxMS0xM10iLCJjaXRhdGlvbnMiOlt7ImJpYmxpb0NvbnRlbnQiOlt7ImVsZWN0cm9uaWNSZXNvdXJjZU51bWJlciI6IjEwLjEzODUvTU86MTk6NDoyNjEiLCJyZWZlcmVuY2VUeXBlIjoiMTciLCJndWlkIjoiNGMwY2E3ZDUtYTNiYy00YTZjLWE4MDktNzA5MjU4N2I3ZmZjIiwidXJsIjpbImh0dHA6Ly9saW5rLnNwcmluZ2VyLmNvbS8xMC4xMzg1L01POjE5OjQ6MjYxIl0sIm51bWJlciI6IjQiLCJ0aXRsZSI6IlBoYXJtYWNva2luZXRpY3Mgb2YgT3hhbGlwbGF0aW4gaW4gSHVtYW5zIiwic2Vjb25kYXJ5VGl0bGUiOiJNZWRpY2FsIE9uY29sb2d5Iiwidm9sdW1lIjoiMTkiLCJhdXRob3JzIjpbIkVocnNzb24sIEguIiwiV2FsbGluLCBJLiIsIllhY2huaW4sIEouIl0sInllYXIiOiIyMDAyIiwicGFnZXMiOiIyNjEtMjY2IiwiZ3JvdXBHdWlkcyI6W10sInJlY29yZFN0YXR1cyI6ImFjdGl2ZSIsInJzeG1sIjoiPHJlY29yZD48cmVmLXR5cGU%2BMTc8L3JlZi10eXBlPjxjb250cmlidXRvcnM%2BPGF1dGhvcnM%2BPGF1dGhvcj48c3R5bGUgc2l6ZT1cIjEwMCVcIiBmb250PVwiZGVmYXVsdFwiPkVocnNzb24sIEguPC9zdHlsZT48L2F1dGhvcj48YXV0aG9yPjxzdHlsZSBzaXplPVwiMTAwJVwiIGZvbnQ9XCJkZWZhdWx0XCI%2BV2FsbGluLCBJLjwvc3R5bGU%2BPC9hdXRob3I%2BPGF1dGhvcj48c3R5bGUgc2l6ZT1cIjEwMCVcIiBmb250PVwiZGVmYXVsdFwiPllhY2huaW4sIEouPC9zdHlsZT48L2F1dGhvcj48L2F1dGhvcnM%2BPC9jb250cmlidXRvcnM%2BPHRpdGxlcz48dGl0bGU%2BPHN0eWxlIHNpemU9XCIxMDAlXCIgZm9udD1cImRlZmF1bHRcIj5QaGFybWFjb2tpbmV0aWNzIG9mIE94YWxpcGxhdGluIGluIEh1bWFuczwvc3R5bGU%2BPC90aXRsZT48c2Vjb25kYXJ5LXRpdGxlPjxzdHlsZSBzaXplPVwiMTAwJVwiIGZvbnQ9XCJkZWZhdWx0XCI%2BTWVkaWNhbCBPbmNvbG9neTwvc3R5bGU%2BPC9zZWNvbmRhcnktdGl0bGU%2BPC90aXRsZXM%2BPGRhdGVzPjx5ZWFyPjxzdHlsZSBzaXplPVwiMTAwJVwiIGZvbnQ9XCJkZWZhdWx0XCI%2BMjAwMjwvc3R5bGU%2BPC95ZWFyPjwvZGF0ZXM%2BPHBhZ2VzPjxzdHlsZSBzaXplPVwiMTAwJVwiIGZvbnQ9XCJkZWZhdWx0XCI%2BMjYxLTI2Njwvc3R5bGU%2BPC9wYWdlcz48dm9sdW1lPjxzdHlsZSBzaXplPVwiMTAwJVwiIGZvbnQ9XCJkZWZhdWx0XCI%2BMTk8L3N0eWxlPjwvdm9sdW1lPjx1cmxzPjxyZWxhdGVkLXVybHM%2BPHVybD48c3R5bGUgc2l6ZT1cIjEwMCVcIiBmb250PVwiZGVmYXVsdFwiPmh0dHA6Ly9saW5rLnNwcmluZ2VyLmNvbS8xMC4xMzg1L01POjE5OjQ6MjYxPC9zdHlsZT48L3VybD48L3JlbGF0ZWQtdXJscz48L3VybHM%2BPGVsZWN0cm9uaWMtcmVzb3VyY2UtbnVtPjxzdHlsZSBzaXplPVwiMTAwJVwiIGZvbnQ9XCJkZWZhdWx0XCI%2BMTAuMTM4NS9NTzoxOTo0OjI2MTwvc3R5bGU%2BPC9lbGVjdHJvbmljLXJlc291cmNlLW51bT48bnVtYmVyPjxzdHlsZSBzaXplPVwiMTAwJVwiIGZvbnQ9XCJkZWZhdWx0XCI%2BNDwvc3R5bGU%2BPC9udW1iZXI%2BPHJlYy1ndWlkPjRjMGNhN2Q1LWEzYmMtNGE2Yy1hODA5LTcwOTI1ODdiN2ZmYzwvcmVjLWd1aWQ%2BPHJlYy11c24%2BMzc8L3JlYy11c24%2BPC9yZWNvcmQ%2BIn1dLCJndWlkIjoiNGMwY2E3ZDUtYTNiYy00YTZjLWE4MDktNzA5MjU4N2I3ZmZjIiwicmVjb3JkIjp7InBhZ2VzIjoiMjYxLTI2NiIsImRhdGVzIjp7InllYXIiOiIyMDAyIn0sInJlZi10eXBlIjoiMTciLCJyZWMtdXNuIjoiMzciLCJudW1iZXIiOiI0IiwicmVjLWd1aWQiOiI0YzBjYTdkNS1hM2JjLTRhNmMtYTgwOS03MDkyNTg3YjdmZmMiLCJ1cmxzIjp7InJlbGF0ZWQtdXJscyI6eyJ1cmwiOiJodHRwOi8vbGluay5zcHJpbmdlci5jb20vMTAuMTM4NS9NTzoxOTo0OjI2MSJ9fSwiY29udHJpYnV0b3JzIjp7ImF1dGhvcnMiOnsiYXV0aG9yIjpbIkVocnNzb24sIEguIiwiV2FsbGluLCBJLiIsIllhY2huaW4sIEouIl19fSwidGl0bGVzIjp7InRpdGxlIjoiUGhhcm1hY29raW5ldGljcyBvZiBPeGFsaXBsYXRpbiBpbiBIdW1hbnMiLCJzZWNvbmRhcnktdGl0bGUiOiJNZWRpY2FsIE9uY29sb2d5In0sInZvbHVtZSI6IjE5IiwiZWxlY3Ryb25pYy1yZXNvdXJjZS1udW0iOiIxMC4xMzg1L01POjE5OjQ6MjYxIn19LHsicmVjb3JkIjp7ImNvbnRyaWJ1dG9ycyI6eyJhdXRob3JzIjp7ImF1dGhvciI6WyJMw6l2aSwgRnJhbmNpcyIsIk1ldHpnZXIsIEfDqXJhcmQiLCJNYXNzYXJpLCBDbGFpcmUiLCJNaWxhbm8sIEfDqXJhcmQiLCJMw6l2aSwgRnJhbmNpcyIsIk1ldHpnZXIsIEfDqXJhcmQiLCJNYXNzYXJpLCBDbGFpcmUiLCJNaWxhbm8sIEfDqXJhcmQiXX19LCJlbGVjdHJvbmljLXJlc291cmNlLW51bSI6IjEwLjIxNjUvMDAwMDMwODgtMjAwMDM4MDEwLTAwMDAxIiwidm9sdW1lIjoiMzgiLCJpc2JuIjoiMTE3OS0xOTI2IiwibnVtYmVyIjoiMSIsInJlZi10eXBlIjoiMTciLCJkYXRlcyI6eyJ5ZWFyIjoiMjAxMiJ9LCJyZWMtZ3VpZCI6IjgyMTI2YzI2LTg0MTUtNDhkYi1iMzEzLTA3ZGVlMmQ5Mzg5YyIsInJlYy11c24iOiI0MjkiLCJ0aXRsZXMiOnsidGl0bGUiOiJPeGFsaXBsYXRpbiIsInNlY29uZGFyeS10aXRsZSI6IkNsaW5pY2FsIFBoYXJtYWNva2luZXRpY3MifSwidXJscyI6eyJyZWxhdGVkLXVybHMiOnsidXJsIjoiaHR0cHM6Ly9saW5rLnNwcmluZ2VyLmNvbS9hcnRpY2xlLzEwLjIxNjUvMDAwMDMwODgtMjAwMDM4MDEwLTAwMDAxIn19LCJhYnN0cmFjdCI6IkNsaW5pY2FsIFBoYXJtYWNva2luZXRpY3MgLSBPeGFsaXBsYXRpbiBpcyB0aGUgZmlyc3QgY2xpbmljYWxseSBhdmFpbGFibGUgZGlhbWlub2N5Y2xvaGV4YW5lIHBsYXRpbnVtIGNvb3JkaW5hdGlvbiBjb21wbGV4LiBUaGUgZHJ1ZyBpcyBub24tY3Jvc3MtcmVzaXN0YW50IHdpdGggY2lzcGxhdGluIG9yIGNhcmJvcGxhdGluIGFuZCBpcyBvbmUuLi4ifSwiZ3VpZCI6IjgyMTI2YzI2LTg0MTUtNDhkYi1iMzEzLTA3ZGVlMmQ5Mzg5YyIsImJpYmxpb0NvbnRlbnQiOlt7Imd1aWQiOiI4MjEyNmMyNi04NDE1LTQ4ZGItYjMxMy0wN2RlZTJkOTM4OWMiLCJ0aXRsZSI6Ik94YWxpcGxhdGluIiwibnVtYmVyIjoiMSIsInJlY29yZFN0YXR1cyI6ImFjdGl2ZSIsInZvbHVtZSI6IjM4IiwiYXV0aG9ycyI6WyJMw6l2aSwgRnJhbmNpcyIsIk1ldHpnZXIsIEfDqXJhcmQiLCJNYXNzYXJpLCBDbGFpcmUiLCJNaWxhbm8sIEfDqXJhcmQiLCJMw6l2aSwgRnJhbmNpcyIsIk1ldHpnZXIsIEfDqXJhcmQiLCJNYXNzYXJpLCBDbGFpcmUiLCJNaWxhbm8sIEfDqXJhcmQiXSwiaXNibiI6IjExNzktMTkyNiIsInllYXIiOiIyMDEyIiwidXJsIjpbImh0dHBzOi8vbGluay5zcHJpbmdlci5jb20vYXJ0aWNsZS8xMC4yMTY1LzAwMDAzMDg4LTIwMDAzODAxMC0wMDAwMSJdLCJyZWZlcmVuY2VUeXBlIjoiMTciLCJlbGVjdHJvbmljUmVzb3VyY2VOdW1iZXIiOiIxMC4yMTY1LzAwMDAzMDg4LTIwMDAzODAxMC0wMDAwMSIsImdyb3VwR3VpZHMiOltdLCJzZWNvbmRhcnlUaXRsZSI6IkNsaW5pY2FsIFBoYXJtYWNva2luZXRpY3MiLCJyc3htbCI6IjxyZWNvcmQ%2BPHJlZi10eXBlPjE3PC9yZWYtdHlwZT48Y29udHJpYnV0b3JzPjxhdXRob3JzPjxhdXRob3I%2BPHN0eWxlIHNpemU9XCIxMDAlXCIgZm9udD1cImRlZmF1bHRcIj5Mw6l2aSwgRnJhbmNpczwvc3R5bGU%2BPC9hdXRob3I%2BPGF1dGhvcj48c3R5bGUgc2l6ZT1cIjEwMCVcIiBmb250PVwiZGVmYXVsdFwiPk1ldHpnZXIsIEfDqXJhcmQ8L3N0eWxlPjwvYXV0aG9yPjxhdXRob3I%2BPHN0eWxlIHNpemU9XCIxMDAlXCIgZm9udD1cImRlZmF1bHRcIj5NYXNzYXJpLCBDbGFpcmU8L3N0eWxlPjwvYXV0aG9yPjxhdXRob3I%2BPHN0eWxlIHNpemU9XCIxMDAlXCIgZm9udD1cImRlZmF1bHRcIj5NaWxhbm8sIEfDqXJhcmQ8L3N0eWxlPjwvYXV0aG9yPjxhdXRob3I%2BPHN0eWxlIHNpemU9XCIxMDAlXCIgZm9udD1cImRlZmF1bHRcIj5Mw6l2aSwgRnJhbmNpczwvc3R5bGU%2BPC9hdXRob3I%2BPGF1dGhvcj48c3R5bGUgc2l6ZT1cIjEwMCVcIiBmb250PVwiZGVmYXVsdFwiPk1ldHpnZXIsIEfDqXJhcmQ8L3N0eWxlPjwvYXV0aG9yPjxhdXRob3I%2BPHN0eWxlIHNpemU9XCIxMDAlXCIgZm9udD1cImRlZmF1bHRcIj5NYXNzYXJpLCBDbGFpcmU8L3N0eWxlPjwvYXV0aG9yPjxhdXRob3I%2BPHN0eWxlIHNpemU9XCIxMDAlXCIgZm9udD1cImRlZmF1bHRcIj5NaWxhbm8sIEfDqXJhcmQ8L3N0eWxlPjwvYXV0aG9yPjwvYXV0aG9ycz48L2NvbnRyaWJ1dG9ycz48dGl0bGVzPjx0aXRsZT48c3R5bGUgc2l6ZT1cIjEwMCVcIiBmb250PVwiZGVmYXVsdFwiPk94YWxpcGxhdGluPC9zdHlsZT48L3RpdGxlPjxzZWNvbmRhcnktdGl0bGU%2BPHN0eWxlIHNpemU9XCIxMDAlXCIgZm9udD1cImRlZmF1bHRcIj5DbGluaWNhbCBQaGFybWFjb2tpbmV0aWNzPC9zdHlsZT48L3NlY29uZGFyeS10aXRsZT48L3RpdGxlcz48ZGF0ZXM%2BPHllYXI%2BPHN0eWxlIHNpemU9XCIxMDAlXCIgZm9udD1cImRlZmF1bHRcIj4yMDEyPC9zdHlsZT48L3llYXI%2BPC9kYXRlcz48dm9sdW1lPjxzdHlsZSBzaXplPVwiMTAwJVwiIGZvbnQ9XCJkZWZhdWx0XCI%2BMzg8L3N0eWxlPjwvdm9sdW1lPjxpc2JuPjxzdHlsZSBzaXplPVwiMTAwJVwiIGZvbnQ9XCJkZWZhdWx0XCI%2BMTE3OS0xOTI2PC9zdHlsZT48L2lzYm4%2BPGFic3RyYWN0PjxzdHlsZSBzaXplPVwiMTAwJVwiIGZvbnQ9XCJkZWZhdWx0XCI%2BQ2xpbmljYWwgUGhhcm1hY29raW5ldGljcyAtIE94YWxpcGxhdGluIGlzIHRoZSBmaXJzdCBjbGluaWNhbGx5IGF2YWlsYWJsZSBkaWFtaW5vY3ljbG9oZXhhbmUgcGxhdGludW0gY29vcmRpbmF0aW9uIGNvbXBsZXguIFRoZSBkcnVnIGlzIG5vbi1jcm9zcy1yZXNpc3RhbnQgd2l0aCBjaXNwbGF0aW4gb3IgY2FyYm9wbGF0aW4gYW5kIGlzIG9uZS4uLjwvc3R5bGU%2BPC9hYnN0cmFjdD48dXJscz48cmVsYXRlZC11cmxzPjx1cmw%2BPHN0eWxlIHNpemU9XCIxMDAlXCIgZm9udD1cImRlZmF1bHRcIj5odHRwczovL2xpbmsuc3ByaW5nZXIuY29tL2FydGljbGUvMTAuMjE2NS8wMDAwMzA4OC0yMDAwMzgwMTAtMDAwMDE8L3N0eWxlPjwvdXJsPjwvcmVsYXRlZC11cmxzPjwvdXJscz48ZWxlY3Ryb25pYy1yZXNvdXJjZS1udW0%2BPHN0eWxlIHNpemU9XCIxMDAlXCIgZm9udD1cImRlZmF1bHRcIj4xMC4yMTY1LzAwMDAzMDg4LTIwMDAzODAxMC0wMDAwMTwvc3R5bGU%2BPC9lbGVjdHJvbmljLXJlc291cmNlLW51bT48bnVtYmVyPjxzdHlsZSBzaXplPVwiMTAwJVwiIGZvbnQ9XCJkZWZhdWx0XCI%2BMTwvc3R5bGU%2BPC9udW1iZXI%2BPHJlYy1ndWlkPjgyMTI2YzI2LTg0MTUtNDhkYi1iMzEzLTA3ZGVlMmQ5Mzg5YzwvcmVjLWd1aWQ%2BPHJlYy11c24%2BNDI5PC9yZWMtdXNuPjwvcmVjb3JkPiJ9XX0seyJyZWNvcmQiOnsiYWJzdHJhY3QiOiJQdXJwb3NlOiBUbyBjaGFyYWN0ZXJpemUgdGhlIHBoYXJtYWNva2luZXRpY3MgYW5kIHBoYXJtYWNvZHluYW1pY3Mgb2Ygb3hhbGlwbGF0aW4gaW4gY2FuY2VyIHBhdGllbnRzIHdpdGggaW1wYWlyZWQgcmVuYWwgZnVuY3Rpb24uIiwidGl0bGVzIjp7InNlY29uZGFyeS10aXRsZSI6IkNsaW5pY2FsIENhbmNlciBSZXNlYXJjaCIsInRpdGxlIjoiT3hhbGlwbGF0aW4gUGhhcm1hY29raW5ldGljcyBhbmQgUGhhcm1hY29keW5hbWljcyBpbiBBZHVsdCBDYW5jZXIgUGF0aWVudHMgd2l0aCBJbXBhaXJlZCBSZW5hbCBGdW5jdGlvbiJ9LCJjb250cmlidXRvcnMiOnsiYXV0aG9ycyI6eyJhdXRob3IiOlsiVGFraW1vdG8sIENocmlzIEguIiwiR3JhaGFtLCBNYXJ0aW4gQS4iLCJMb2Nrd29vZCwgR3JhaGFtIiwiTmcsIENoZWUgTS4iLCJHb2V0eiwgQW5kcmV3IiwiR3JlZW5zbGFkZSwgRGVubmlzIiwiUmVtaWNrLCBTY290IEMuIiwiU2hhcm1hLCBTdW5pbCIsIk1hbmksIFNyaWRoYXIiLCJSYW1hbmF0aGFuLCBSYW1lc2ggSy4iLCJTeW5vbGQsIFRpbW90aHkgVy4iLCJEb3Jvc2hvdywgSmFtZXMgSC4iLCJIYW1pbHRvbiwgQW5uZSIsIk11bGtlcmluLCBEYW5pZWwgTC4iLCJJdnksIFBlcmN5IiwiRWdvcmluLCBNZXJyaWxsIEouIiwiR3Jl) | 0.51 μM  (1.1) | 1.1 μM†  (1.0, 0.2) | 2.4 μM†  (1.3, 0.04) | 1.5 μM†  (1.3, 0.1) |
| FOLFIRINOX | 1000%* | [[14, 15]](https://web.endnote.com/citations/eyJkaXNwbGF5VGV4dCI6IlsxNCwgMTVdIiwiY2l0YXRpb25zIjpbeyJyZWNvcmQiOnsicmVjLXVzbiI6IjEwNTciLCJ2b2x1bWUiOiIzNzkiLCJudW1iZXIiOiIyNSIsImVsZWN0cm9uaWMtcmVzb3VyY2UtbnVtIjoiMTAuMTA1Ni9ORUpNb2ExODA5Nzc1IiwiaXNibiI6IjAwMjgtNDc5MyIsImNvbnRyaWJ1dG9ycyI6eyJhdXRob3JzIjp7ImF1dGhvciI6WyJUaGllcnJ5IENvbnJveSIsIlBhc2NhbCBIYW1tZWwiLCJNb2hhbWVkIEhlYmJhciIsIk1laGVyIEJlbiBBYmRlbGdoYW5pIiwiQWxpY2UgQy4gV2VpIiwiSmVhbi1MdWMgUmFvdWwiLCJMYXVyZW5jZSBDaG9uw6kiLCJFcmljIEZyYW5jb2lzIiwiUGFzY2FsIEFydHJ1IiwiSmFtZXMgSi4gQmlhZ2kiLCJUaGllcnJ5IExlY29tdGUiLCJFcmljIEFzc2VuYXQiLCJSb2dlciBGYXJvdXgiLCJNYXJjIFljaG91IiwiSnVsaWVuIFZvbGV0IiwiQWxhaW4gU2F1dmFuZXQiLCJHaWxsZXMgQnJleXNhY2hlciIsIkZyw6lkw6lyaWMgRGkgRmlvcmUiLCJDaHJpc3RpbmUgQ3JpcHBzIiwiUGV0ciBLYXZhbiIsIlBhdHJpY2sgVGV4ZXJlYXUiLCJLYXJpbmUgQm91aGllci1MZXBvcnJpZXIiLCJGYWl6YSBLaGVtaXNzYS1Ba291eiIsIkplYW4tTG91aXMgTGVnb3V4IiwiQsOpYXRhIEp1enluYSIsIlNvcGhpZSBHb3VyZ291IiwiQ2hyaXN0b3BoZXIgSi4gT%2BKAmUNhbGxhZ2hhbiIsIkNsYWlyZSBKb3VmZnJveS1aZWxsZXIiLCJQYXRyaWNrIFJhdCIsIkRhdmlkIE1hbGthIiwiRmxvcmVuY2UgQ2FzdGFuIiwiSmVhbi1CYXB0aXN0ZSBCYWNoZXQiXX19LCJ1cmxzIjp7InJlbGF0ZWQtdXJscyI6eyJ1cmwiOiJodHRwczovL3d3dy5uZWptLm9yZy9kb2kvZnVsbC8xMC4xMDU2L05FSk1vYTE4MDk3NzUifX0sInJlZi10eXBlIjoiMTciLCJkYXRlcyI6eyJ5ZWFyIjoiMjAxOCJ9LCJ0aXRsZXMiOnsidGl0bGUiOiJGT0xGSVJJTk9YIG9yIEdlbWNpdGFiaW5lIGFzIEFkanV2YW50IFRoZXJhcHkgZm9yIFBhbmNyZWF0aWMgQ2FuY2VyIiwic2Vjb25kYXJ5LXRpdGxlIjoiTmV3IEVuZ2xhbmQgSm91cm5hbCBvZiBNZWRpY2luZSJ9LCJhYnN0cmFjdCI6IkFtb25nIHBhdGllbnRzIHdpdGggbWV0YXN0YXRpYyBwYW5jcmVhdGljIGNhbmNlciwgY29tYmluYXRpb24gY2hlbW90aGVyYXB5IHdpdGggZmx1b3JvdXJhY2lsLCBsZXVjb3ZvcmluLCBpcmlub3RlY2FuLCBhbmQgb3hhbGlwbGF0aW4gKEZPTEZJUklOT1gpIGxlYWRzIHRvIGxvbmdlciBvdmVyYWxsIHN1cnZpdmFsIHRoYW4gZ2VtY2l0YWJpbmUgLi4uIiwicmVjLWd1aWQiOiI3ZDJjODlhYi1mMDUxLTQwM2YtOGEyMC1jOTI5ZmNmMDRhZjgifSwiZ3VpZCI6IjdkMmM4OWFiLWYwNTEtNDAzZi04YTIwLWM5MjlmY2YwNGFmOCIsImJpYmxpb0NvbnRlbnQiOlt7InNlY29uZGFyeVRpdGxlIjoiTmV3IEVuZ2xhbmQgSm91cm5hbCBvZiBNZWRpY2luZSIsInVybCI6WyJodHRwczovL3d3dy5uZWptLm9yZy9kb2kvZnVsbC8xMC4xMDU2L05FSk1vYTE4MDk3NzUiXSwieWVhciI6IjIwMTgiLCJyc3htbCI6IjxyZWNvcmQ%2BPHJlZi10eXBlPjE3PC9yZWYtdHlwZT48Y29udHJpYnV0b3JzPjxhdXRob3JzPjxhdXRob3I%2BPHN0eWxlIHNpemU9XCIxMDAlXCIgZm9udD1cImRlZmF1bHRcIj5UaGllcnJ5IENvbnJveTwvc3R5bGU%2BPC9hdXRob3I%2BPGF1dGhvcj48c3R5bGUgc2l6ZT1cIjEwMCVcIiBmb250PVwiZGVmYXVsdFwiPlBhc2NhbCBIYW1tZWw8L3N0eWxlPjwvYXV0aG9yPjxhdXRob3I%2BPHN0eWxlIHNpemU9XCIxMDAlXCIgZm9udD1cImRlZmF1bHRcIj5Nb2hhbWVkIEhlYmJhcjwvc3R5bGU%2BPC9hdXRob3I%2BPGF1dGhvcj48c3R5bGUgc2l6ZT1cIjEwMCVcIiBmb250PVwiZGVmYXVsdFwiPk1laGVyIEJlbiBBYmRlbGdoYW5pPC9zdHlsZT48L2F1dGhvcj48YXV0aG9yPjxzdHlsZSBzaXplPVwiMTAwJVwiIGZvbnQ9XCJkZWZhdWx0XCI%2BQWxpY2UgQy4gV2VpPC9zdHlsZT48L2F1dGhvcj48YXV0aG9yPjxzdHlsZSBzaXplPVwiMTAwJVwiIGZvbnQ9XCJkZWZhdWx0XCI%2BSmVhbi1MdWMgUmFvdWw8L3N0eWxlPjwvYXV0aG9yPjxhdXRob3I%2BPHN0eWxlIHNpemU9XCIxMDAlXCIgZm9udD1cImRlZmF1bHRcIj5MYXVyZW5jZSBDaG9uw6k8L3N0eWxlPjwvYXV0aG9yPjxhdXRob3I%2BPHN0eWxlIHNpemU9XCIxMDAlXCIgZm9udD1cImRlZmF1bHRcIj5FcmljIEZyYW5jb2lzPC9zdHlsZT48L2F1dGhvcj48YXV0aG9yPjxzdHlsZSBzaXplPVwiMTAwJVwiIGZvbnQ9XCJkZWZhdWx0XCI%2BUGFzY2FsIEFydHJ1PC9zdHlsZT48L2F1dGhvcj48YXV0aG9yPjxzdHlsZSBzaXplPVwiMTAwJVwiIGZvbnQ9XCJkZWZhdWx0XCI%2BSmFtZXMgSi4gQmlhZ2k8L3N0eWxlPjwvYXV0aG9yPjxhdXRob3I%2BPHN0eWxlIHNpemU9XCIxMDAlXCIgZm9udD1cImRlZmF1bHRcIj5UaGllcnJ5IExlY29tdGU8L3N0eWxlPjwvYXV0aG9yPjxhdXRob3I%2BPHN0eWxlIHNpemU9XCIxMDAlXCIgZm9udD1cImRlZmF1bHRcIj5FcmljIEFzc2VuYXQ8L3N0eWxlPjwvYXV0aG9yPjxhdXRob3I%2BPHN0eWxlIHNpemU9XCIxMDAlXCIgZm9udD1cImRlZmF1bHRcIj5Sb2dlciBGYXJvdXg8L3N0eWxlPjwvYXV0aG9yPjxhdXRob3I%2BPHN0eWxlIHNpemU9XCIxMDAlXCIgZm9udD1cImRlZmF1bHRcIj5NYXJjIFljaG91PC9zdHlsZT48L2F1dGhvcj48YXV0aG9yPjxzdHlsZSBzaXplPVwiMTAwJVwiIGZvbnQ9XCJkZWZhdWx0XCI%2BSnVsaWVuIFZvbGV0PC9zdHlsZT48L2F1dGhvcj48YXV0aG9yPjxzdHlsZSBzaXplPVwiMTAwJVwiIGZvbnQ9XCJkZWZhdWx0XCI%2BQWxhaW4gU2F1dmFuZXQ8L3N0eWxlPjwvYXV0aG9yPjxhdXRob3I%2BPHN0eWxlIHNpemU9XCIxMDAlXCIgZm9udD1cImRlZmF1bHRcIj5HaWxsZXMgQnJleXNhY2hlcjwvc3R5bGU%2BPC9hdXRob3I%2BPGF1dGhvcj48c3R5bGUgc2l6ZT1cIjEwMCVcIiBmb250PVwiZGVmYXVsdFwiPkZyw6lkw6lyaWMgRGkgRmlvcmU8L3N0eWxlPjwvYXV0aG9yPjxhdXRob3I%2BPHN0eWxlIHNpemU9XCIxMDAlXCIgZm9udD1cImRlZmF1bHRcIj5DaHJpc3RpbmUgQ3JpcHBzPC9zdHlsZT48L2F1dGhvcj48YXV0aG9yPjxzdHlsZSBzaXplPVwiMTAwJVwiIGZvbnQ9XCJkZWZhdWx0XCI%2BUGV0ciBLYXZhbjwvc3R5bGU%2BPC9hdXRob3I%2BPGF1dGhvcj48c3R5bGUgc2l6ZT1cIjEwMCVcIiBmb250PVwiZGVmYXVsdFwiPlBhdHJpY2sgVGV4ZXJlYXU8L3N0eWxlPjwvYXV0aG9yPjxhdXRob3I%2BPHN0eWxlIHNpemU9XCIxMDAlXCIgZm9udD1cImRlZmF1bHRcIj5LYXJpbmUgQm91aGllci1MZXBvcnJpZXI8L3N0eWxlPjwvYXV0aG9yPjxhdXRob3I%2BPHN0eWxlIHNpemU9XCIxMDAlXCIgZm9udD1cImRlZmF1bHRcIj5GYWl6YSBLaGVtaXNzYS1Ba291ejwvc3R5bGU%2BPC9hdXRob3I%2BPGF1dGhvcj48c3R5bGUgc2l6ZT1cIjEwMCVcIiBmb250PVwiZGVmYXVsdFwiPkplYW4tTG91aXMgTGVnb3V4PC9zdHlsZT48L2F1dGhvcj48YXV0aG9yPjxzdHlsZSBzaXplPVwiMTAwJVwiIGZvbnQ9XCJkZWZhdWx0XCI%2BQsOpYXRhIEp1enluYTwvc3R5bGU%2BPC9hdXRob3I%2BPGF1dGhvcj48c3R5bGUgc2l6ZT1cIjEwMCVcIiBmb250PVwiZGVmYXVsdFwiPlNvcGhpZSBHb3VyZ291PC9zdHlsZT48L2F1dGhvcj48YXV0aG9yPjxzdHlsZSBzaXplPVwiMTAwJVwiIGZvbnQ9XCJkZWZhdWx0XCI%2BQ2hyaXN0b3BoZXIgSi4gT%2BKAmUNhbGxhZ2hhbjwvc3R5bGU%2BPC9hdXRob3I%2BPGF1dGhvcj48c3R5bGUgc2l6ZT1cIjEwMCVcIiBmb250PVwiZGVmYXVsdFwiPkNsYWlyZSBKb3VmZnJveS1aZWxsZXI8L3N0eWxlPjwvYXV0aG9yPjxhdXRob3I%2BPHN0eWxlIHNpemU9XCIxMDAlXCIgZm9udD1cImRlZmF1bHRcIj5QYXRyaWNrIFJhdDwvc3R5bGU%2BPC9hdXRob3I%2BPGF1dGhvcj48c3R5bGUgc2l6ZT1cIjEwMCVcIiBmb250PVwiZGVmYXVsdFwiPkRhdmlkIE1hbGthPC9zdHlsZT48L2F1dGhvcj48YXV0aG9yPjxzdHlsZSBzaXplPVwiMTAwJVwiIGZvbnQ9XCJkZWZhdWx0XCI%2BRmxvcmVuY2UgQ2FzdGFuPC9zdHlsZT48L2F1dGhvcj48YXV0aG9yPjxzdHlsZSBzaXplPVwiMTAwJVwiIGZvbnQ9XCJkZWZhdWx0XCI%2BSmVhbi1CYXB0aXN0ZSBCYWNoZXQ8L3N0eWxlPjwvYXV0aG9yPjwvYXV0aG9ycz48L2NvbnRyaWJ1dG9ycz48dGl0bGVzPjx0aXRsZT48c3R5bGUgc2l6ZT1cIjEwMCVcIiBmb250PVwiZGVmYXVsdFwiPkZPTEZJUklOT1ggb3IgR2VtY2l0YWJpbmUgYXMgQWRqdXZhbnQgVGhlcmFweSBmb3IgUGFuY3JlYXRpYyBDYW5jZXI8L3N0eWxlPjwvdGl0bGU%2BPHNlY29uZGFyeS10aXRsZT48c3R5bGUgc2l6ZT1cIjEwMCVcIiBmb250PVwiZGVmYXVsdFwiPk5ldyBFbmdsYW5kIEpvdXJuYWwgb2YgTWVkaWNpbmU8L3N0eWxlPjwvc2Vjb25kYXJ5LXRpdGxlPjwvdGl0bGVzPjxkYXRlcz48eWVhcj48c3R5bGUgc2l6ZT1cIjEwMCVcIiBmb250PVwiZGVmYXVsdFwiPjIwMTg8L3N0eWxlPjwveWVhcj48L2RhdGVzPjx2b2x1bWU%2BPHN0eWxlIHNpemU9XCIxMDAlXCIgZm9udD1cImRlZmF1bHRcIj4zNzk8L3N0eWxlPjwvdm9sdW1lPjxpc2JuPjxzdHlsZSBzaXplPVwiMTAwJVwiIGZvbnQ9XCJkZWZhdWx0XCI%2BMDAyOC00NzkzPC9zdHlsZT48L2lzYm4%2BPGFic3RyYWN0PjxzdHlsZSBzaXplPVwiMTAwJVwiIGZvbnQ9XCJkZWZhdWx0XCI%2BQW1vbmcgcGF0aWVudHMgd2l0aCBtZXRhc3RhdGljIHBhbmNyZWF0aWMgY2FuY2VyLCBjb21iaW5hdGlvbiBjaGVtb3RoZXJhcHkgd2l0aCBmbHVvcm91cmFjaWwsIGxldWNvdm9yaW4sIGlyaW5vdGVjYW4sIGFuZCBveGFsaXBsYXRpbiAoRk9MRklSSU5PWCkgbGVhZHMgdG8gbG9uZ2VyIG92ZXJhbGwgc3Vydml2YWwgdGhhbiBnZW1jaXRhYmluZSAuLi48L3N0eWxlPjwvYWJzdHJhY3Q%2BPHVybHM%2BPHJlbGF0ZWQtdXJscz48dXJsPjxzdHlsZSBzaXplPVwiMTAwJVwiIGZvbnQ9XCJkZWZhdWx0XCI%2BaHR0cHM6Ly93d3cubmVqbS5vcmcvZG9pL2Z1bGwvMTAuMTA1Ni9ORUpNb2ExODA5Nzc1PC9zdHlsZT48L3VybD48L3JlbGF0ZWQtdXJscz48L3VybHM%2BPGVsZWN0cm9uaWMtcmVzb3VyY2UtbnVtPjxzdHlsZSBzaXplPVwiMTAwJVwiIGZvbnQ9XCJkZWZhdWx0XCI%2BMTAuMTA1Ni9ORUpNb2ExODA5Nzc1PC9zdHlsZT48L2VsZWN0cm9uaWMtcmVzb3VyY2UtbnVtPjxudW1iZXI%2BPHN0eWxlIHNpemU9XCIxMDAlXCIgZm9udD1cImRlZmF1bHRcIj4yNTwvc3R5bGU%2BPC9udW1iZXI%2BPHJlYy1ndWlkPjdkMmM4OWFiLWYwNTEtNDAzZi04YTIwLWM5MjlmY2YwNGFmODwvcmVjLWd1aWQ%2BPHJlYy11c24%2BMTA1NzwvcmVjLXVzbj48L3JlY29yZD4iLCJndWlkIjoiN2QyYzg5YWItZjA1MS00MDNmLThhMjAtYzkyOWZjZjA0YWY4IiwiZWxlY3Ryb25pY1Jlc291cmNlTnVtYmVyIjoiMTAuMTA1Ni9ORUpNb2ExODA5Nzc1IiwicmVjb3JkU3RhdHVzIjoiYWN0aXZlIiwidGl0bGUiOiJGT0xGSVJJTk9YIG9yIEdlbWNpdGFiaW5lIGFzIEFkanV2YW50IFRoZXJhcHkgZm9yIFBhbmNyZWF0aWMgQ2FuY2VyIiwiaXNibiI6IjAwMjgtNDc5MyIsInJlZmVyZW5jZVR5cGUiOiIxNyIsInZvbHVtZSI6IjM3OSIsImF1dGhvcnMiOlsiVGhpZXJyeSBDb25yb3kiLCJQYXNjYWwgSGFtbWVsIiwiTW9oYW1lZCBIZWJiYXIiLCJNZWhlciBCZW4gQWJkZWxnaGFuaSIsIkFsaWNlIEMuIFdlaSIsIkplYW4tTHVjIFJhb3VsIiwiTGF1cmVuY2UgQ2hvbsOpIiwiRXJpYyBGcmFuY29pcyIsIlBhc2NhbCBBcnRydSIsIkphbWVzIEouIEJpYWdpIiwiVGhpZXJyeSBMZWNvbXRlIiwiRXJpYyBBc3NlbmF0IiwiUm9nZXIgRmFyb3V4IiwiTWFyYyBZY2hvdSIsIkp1bGllbiBWb2xldCIsIkFsYWluIFNhdXZhbmV0IiwiR2lsbGVzIEJyZXlzYWNoZXIiLCJGcsOpZMOpcmljIERpIEZpb3JlIiwiQ2hyaXN0aW5lIENyaXBwcyIsIi4uLiIsIkplYW4tQmFwdGlzdGUgQmFjaGV0Il0sImdyb3VwR3VpZHMiOltdLCJudW1iZXIiOiIyNSJ9XX0seyJndWlkIjoiYjIzYzc5MjYtOTJmNS00YmU0LTg4NjYtNmQwZDg0ZmJkOTNiIiwicmVjb3JkIjp7ImNvbnRyaWJ1dG9ycyI6eyJhdXRob3JzIjp7ImF1dGhvciI6WyJSZWJlY2NhIEwuIFBvcnRlci) | 0.045%  (0.006) | 0.032%  (0.05, 0.9) | 0.14%  (0.1, 0.2) | 0.35%†  (0.1,0.004) |

*IC_50_ values were interpolated from the graphs (Fig. 1, 6  and Suppl. Fig. 3, with the curves plotted as non-linear fit. FOLFIRINOX is a combination treatment consisting of folinic acid, 5-fluorouracil, irinotecan, and oxaliplatin. The maximum treatment concentration (100%) is equal to 0.428 μM folinic acid, 34.4 μM 5-fluorouracil, 0.4 μM SN-38 (active compound of irinotecan), and 0.32 μM oxaliplatin. The C_max_ is the average maximum plasma concentration as reported in literature. † indicates a significantly altered IC_50_ compared to the parental cells as determined by Student’s T-test.*


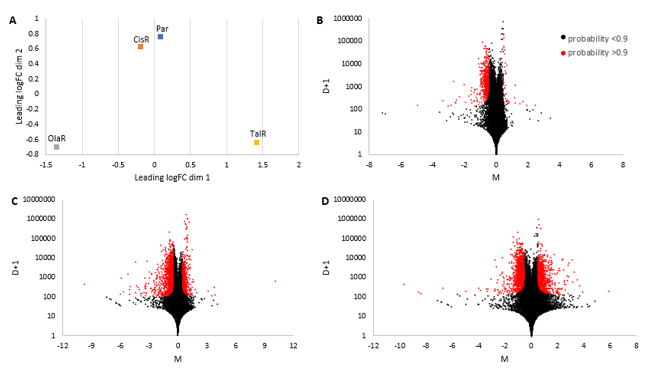


***Supplemental figure 1. Similarities in gene expression between Capan-1 parental and resistant lines. Based on RNA sequencing data.*** *A) Multidimensional scaling plot. B) Mean-difference plot for Capan-1CisR. C) Mean-difference plot for Capan-1OlaR. D) Mean-difference plot for Capan-1TalR. (n=1).*


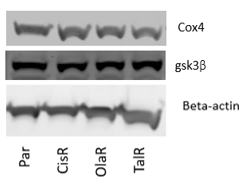


***Supplemental figure 2. Protein expression of Cox4 and Gsk3beta as measured by Western blotting.*** *The bands for beta-actin are duplicated from figure 3, with the addition of the previously excluded CisR sample.*


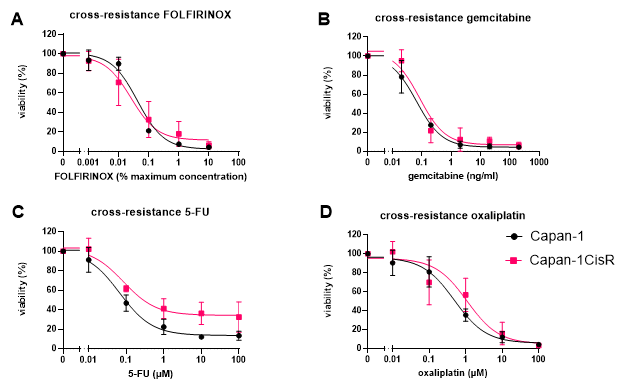


***Supplemental figure 3.* V*iability assay in parental and resistant cells after seven days of treatment as measured by PrestoBlue staining (n=3)***


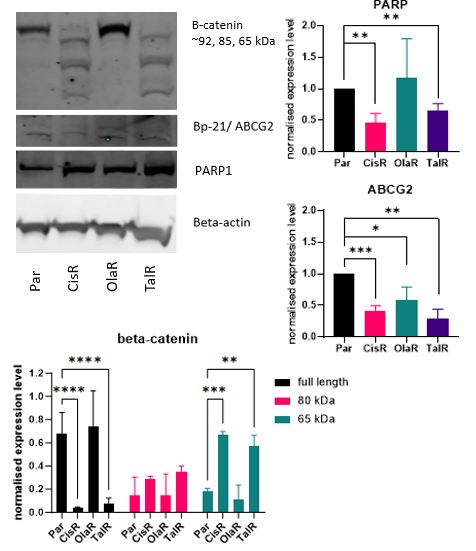

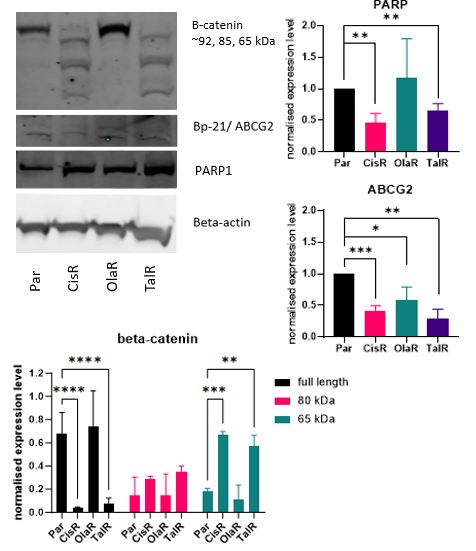


***Supplemental figure 4. Expression of beta-catenin, PARP1 and ABCG2 in Capan-1 by Western blot.*** *This figure is an extended version of figure 3 which includes the previously excluded CisR sample. Expression was quantified using ImageJ and corrected against the loading control beta-actin and then normalised against the parental cells. Student’s T-test was used to identify statistical differences between each resistant cell line and the parental cell line. Error bars show the standard deviation of the mean (n=3). *:p<0.05, **:p<0.005, ***:p<0.001.*

**References supplemental data**

[1. Rajkumar, P., et al., *Cisplatin Concentrations in Long and Short Duration Infusion: Implications for the Optimal Time of Radiation Delivery.* Journal of Clinical and Diagnostic Research : JCDR, 2016. **10**(7).](https://web.endnote.com/reference-list/)

[2. AstraZeneca, A.B., *CHMP assessment report Lynparza*. 2014: Södertälje. p. 187-187.](https://web.endnote.com/reference-list/)

[3. AstraZeneca, A.B., *NDA/BLA Multi-disciplinary Review and Evaluation NDA 208558 LynparzaTM (Olaparib)*. 2017: Södertälje. p. 233-233.](https://web.endnote.com/reference-list/)

[4. Luo, Y., et al., *Pharmacokinetics, safety, and antitumor activity of talazoparib monotherapy in Chinese patients with advanced solid tumors.* Investigational New Drugs, 2023. **41**(3): p. 503-511.](https://web.endnote.com/reference-list/)

[5. Naito, Y., et al., *Safety, pharmacokinetics, and preliminary efficacy of the PARP inhibitor talazoparib in Japanese patients with advanced solid tumors: phase 1 study.* Investigational New Drugs, 2021. **39**(6): p. 1568-1576.](https://web.endnote.com/reference-list/)

[6. Pfizer Europe, M.E., *Assessment report Talzenna*. 2019: Amsterdam. p. 140-140.](https://web.endnote.com/reference-list/)

[7. Caffo, O., et al., *Pharmacokinetic study of gemcitabine, given as prolonged infusion at fixed dose rate, in combination with cisplatin in patients with advanced non-small-cell lung cancer.* Cancer Chemotherapy and Pharmacology, 2010. **65**(6): p. 1197-1202.](https://web.endnote.com/reference-list/)

[8. Masumori, N., et al., *Measurement of Plasma Concentration of Gemcitabine and Its Metabolite dFdU in Hemodialysis Patients with Advanced Urothelial Cancer.* Japanese Journal of Clinical Oncology, 2008. **38**(3): p. 182-185.](https://web.endnote.com/reference-list/)

[9. Casale, F., et al., *Plasma concentrations of 5-fluorouracil and its metabolites in colon cancer patients.* Pharmacological research, 2004. **50**(2).](https://web.endnote.com/reference-list/)

[10. Di Paolo, A., et al., *5-Fluorouracil Pharmacokinetics Predicts Disease-free Survival in Patients Administered Adjuvant Chemotherapy for Colorectal Cancer.* Clinical Cancer Research, 2008. **14**(9): p. 2749-2755.](https://web.endnote.com/reference-list/)

[11. Ehrsson, H., I. Wallin, and J. Yachnin, *Pharmacokinetics of Oxaliplatin in Humans.* Medical Oncology, 2002. **19**(4): p. 261-266.](https://web.endnote.com/reference-list/)

[12. Lévi, F., et al., *Oxaliplatin.* Clinical Pharmacokinetics, 2012. **38**(1).](https://web.endnote.com/reference-list/)

[13. Takimoto, C.H., et al., *Oxaliplatin Pharmacokinetics and Pharmacodynamics in Adult Cancer Patients with Impaired Renal Function.* Clinical Cancer Research, 2007. **13**(16): p. 4832-4839.](https://web.endnote.com/reference-list/)

[14. Conroy, T., et al., *FOLFIRINOX or Gemcitabine as Adjuvant Therapy for Pancreatic Cancer.* New England Journal of Medicine, 2018. **379**(25).](https://web.endnote.com/reference-list/)

[15. Porter, R.L., et al., *Epithelial to mesenchymal plasticity and differential response to therapies in pancreatic ductal adenocarcinoma.* Proceedings of the National Academy of Sciences, 2019. **116**(52).](https://web.endnote.com/reference-list/)
